# Forkhead box versus NF-κB hippocampal snRNA-seq profiles distinguish anti-Drebrin- and anti-GAD65-positive encephalitis

**DOI:** 10.1186/s12974-026-03951-8

**Published:** 2026-07-07

**Authors:** Karen M.J. van Loo, Daniel S. Galvis-Montes, Annika Breuer, Juliane L. Berns, Chiara A. Hummel, Tobias Baumgartner, Moritz Freyberg, Katharina M. Mair, Jan Bauer, Theodor Rüber, Ashley J. van Waardenberg, Motaz Hamed, Valeri Borger, Hartmut Vatter, Rainer Surges, Susanne Schoch, Albert J. Becker, Julika Pitsch

**Affiliations:** 1 Department of Epileptology, Neurology, RWTH Aachen University, Aachen, Germany; 2 Department of Epileptology, University Hospital Bonn, Venusberg-Campus 1, Bonn, D-53127 Germany; 3 Department of Neuroimmunology, Centre for Brain Research, Medical University of Vienna, Vienna, Austria; 4 Department of Neuroradiology, University Hospital Bonn, Bonn, Germany; 5 i-Synapse, Cairns, Australia; 6 Clinic for Neurosurgery, University Hospital Bonn, Bonn, Germany; 7 Institute of Cellular Neurosciences II, University Hospital Bonn, Bonn, Germany

**Keywords:** Autoimmune encephalitis, Glutamate decarboxylase 65 (GAD65), Temporal lobe epilepsy, FoxO signaling pathway, Drebrin

## Abstract

**Background and objectives:**

Autoantibodies (ABs) against intracellular proteins, including glutamate-decarboxylase 65 (anti-GAD65), are increasingly recognized in autoimmune and limbic encephalitis (AE/LE). Anti-GAD65 LE frequently progresses to severe temporal lobe epilepsy (TLE), neuropathologically characterized by hippocampal sclerosis (HS) and variable infiltration of cytotoxic T lymphocytes (CTLs). Recently, we have identified Drebrin (DBN) as a new intracellular target protein of ABs in index patients with suspected AE. Here, we aim to characterize key molecular and cellular signatures of hippocampal tissue from anti-GAD65- (GAD65-TLE) versus anti-DBN-positive TLE (DBN-TLE) patients correlated to clinical parameters.

**Methods:**

We examined hippocampal neuropathology and performed exploratory single-nucleus RNA sequencing (snRNA-seq) of hippocampal tissue from DBN- and GAD65-TLE patients, integrated with key clinical data from a large patient cohort.

**Results:**

Although the hippocampi of the two patient groups were neuropathologically virtually indistinguishable, exploratory snRNA-seq revealed distinct transcriptional programs. DBN-TLE patients (*n* = 2) showed transcriptional signatures enriched for forkhead box (Fox) transcription factor family, whereas GAD65-TLE patients (*n* = 2) displayed transcriptional signatures enriched for transcripts related to NF-κB- signaling. In a larger cohort, DBN-TLE patients (*n* = 22) showed significantly more favorable pharmacological responsiveness than GAD65-TLE patients (*n* = 35), who were largely pharmacoresistant. Notably, in a T cell-mediated mouse model for LE, similar inflammatory programs were dynamically regulated.

**Conclusion:**

These findings provide a discovery-based transcriptomic signatures of rare autoimmune hippocampal tissue, revealing distinct immune-associated transcriptional states in anti-DBN- versus anti-GAD65-positive AE/TLE patients despite virtually indistinguishable hippocampal pathology in both groups and support further investigations of disease-specific therapeutic strategies.

**Supplementary Information:**

The online version contains supplementary material available at 10.1186/s12974-026-03951-8.

## Introduction

Autoantibody (AB)-associated autoimmune encephalitis (AE) often affects limbic structures, with seizures typically originating in the hippocampal formation, a condition referred to as limbic encephalitis (LE). LE is a major cause of new-onset temporal lobe epilepsy (TLE) in adult patients [1, 2] and is frequently associated with segmental neuronal loss and concomitant gliosis in the hippocampus, i.e. hippocampal sclerosis (HS) [3].

In a large study from the Department of Epileptology at the University Hospital Bonn, ABs targeting the Glutamate decarboxylase (GAD) 65 (anti-GAD65) were most commonly found in adult TLE patients with new-onset TLE and suspected AE [1]. However, key clinical parameters of TLE associated with anti-GAD65 (GAD65-TLE) correlate rather poorly with anti-GAD65 titers [4]. Neuropathologically, prominent cytotoxic T cells (CTLs) present in HS brain biopsies of patients with anti-GAD65 have been associated with hippocampal tissue injury. CTL numbers correlate with neuronal rarefication rather than epileptic activity [5]. GAD65 is an intracellular enzyme that converts the neurotransmitter glutamate into Gamma-aminobutyric acid (GABA) [6]. The functional relevance of GAD65 ABs in the pathogenesis of AE remains controversial. Recent data show that GAD65 ABs inhibit enzymatic GAD65 activity [6] and increase network activity in-vitro [7], while some studies report no significant anti-GAD65 effect [8, 9]. The relative pathogenetic dynamics of adaptive and innate immune mechanisms remain vague. This includes limited understanding of the molecular cascades active in different immune cell compartments. Such immunological ambiguities are observed not only in GAD65-TLE but also in other AE syndromes associated with *intracellular* ABs, which are increasingly reported (for review see [2, 10]).

In this context, we have recently observed that ABs targeting the intracellular epitopes of the protein Drebrin (anti-DBN) are associated with a clinical syndrome of seizures and neuropsychiatric impairment, as well as chronic inflammation of limbic and occasionally extralimbic structures [11]. Drebrin is a structural element of neurites and the excitatory postsynapse highly relevant for dendritic spine scaffolding [12, 13], and has been suggested as protective against electrically-induced seizures [14]. Functional analyses indicated increased neuronal activity following anti-DBN exposure, potentially associated with disturbed dendritic spine function enforcing and altered synaptic coupling [11]. A rarely available neocortical brain biopsy from an anti-DBN-positive TLE (DBN-TLE) patient showed neuropathological evidence of scattered CD8^+^ T cell infiltrates in the cortical grey matter consistent with neurodegeneration. A comprehensive DBN-TLE cohort combining molecular profiling with detailed clinical data has so far been lacking, limiting mechanistic insight into this disease subtype.

Surgical biopsies of anti-GAD65 as well as also anti-DBN patients with epilepsy are exceptionally rare [3, 10, 15, 16]. Leveraging neuroanatomically well-preserved hippocampal specimens from these rare cases, we investigated whether AE/TLE associated with antibodies against the intracellular proteins GAD65 and Drebrin represents a biologically overlapping inflammatory condition or whether these entities are associated with divergent inflammatory and transcriptional signatures. To address this, we performed integrated analyses of rare hippocampal tissue from patients with anti-DBN- or anti-GAD65-associated TLE, including neuropathological assessment and single-nucleus RNA sequencing (snRNA-seq), together with clinical metadata such as treatment response. To provide a complementary experimental inflammatory reference framework, we additionally examined a mouse model based on hippocampal expression of ovalbumin (OVA) in transgenic mice carrying OVA-specific CD8^+^ T cells. This model captures defined aspects of CD8^+^ T cell–mediated neuroinflammation and enables comparison with key immune-associated transcriptional programs observed in human AE-associated TLE.

## Results

### cMRI indicates fundamental involvement of limbic structures in DBN- and GAD65-TLE

**Fig. 1:**
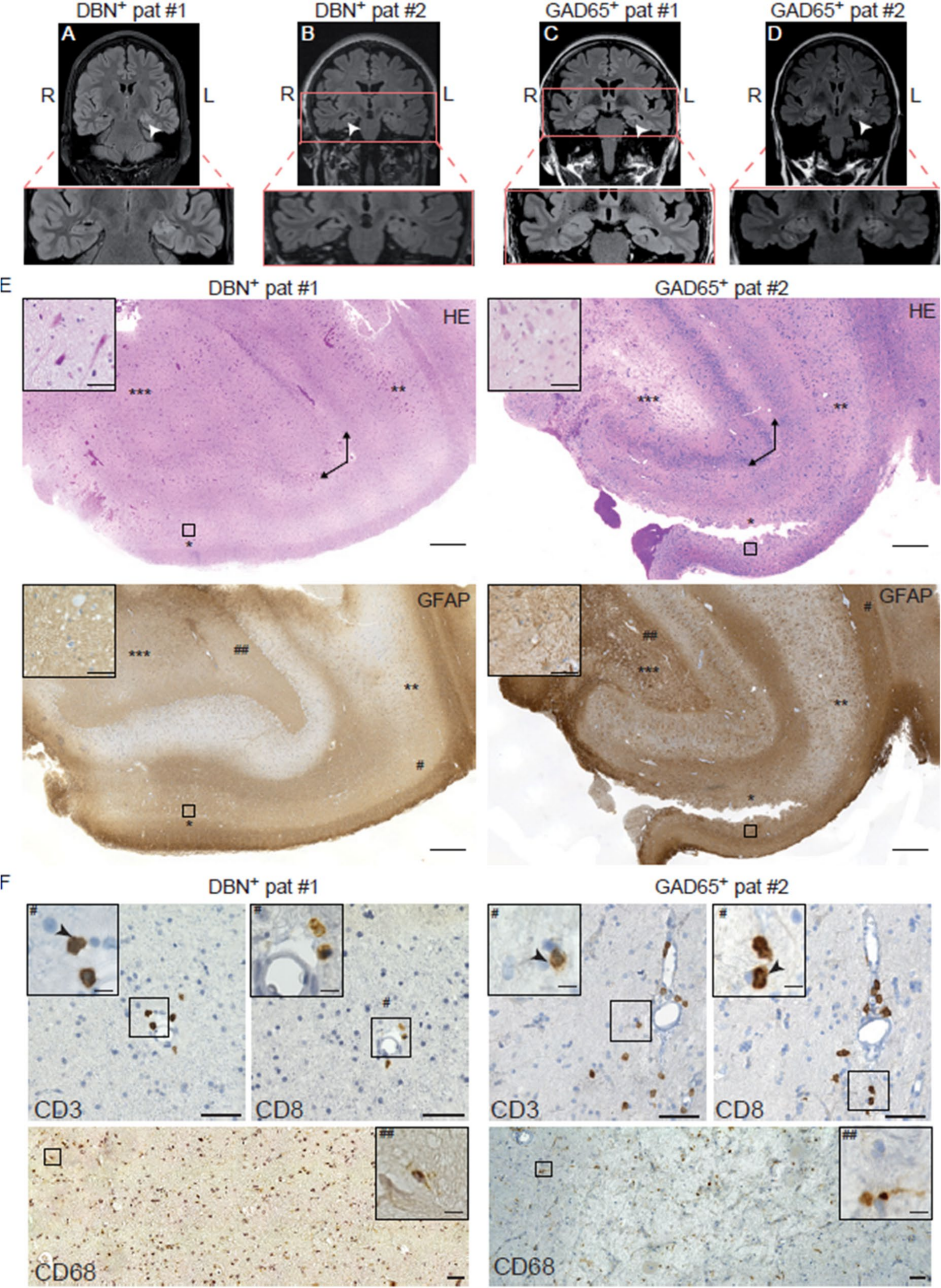
MR imaging and neuropathological manifestations of DBN- and GAD65-TLE patients. **A**-**D** Coronal T2-weighted fluid-attenuated inversion recovery (FLAIR) brain MRI sequences show (**A**) hippocampal sclerosis on the left (white arrow), or (**B**) on the right side in anti-DBN-positive patients (DBN^+^, white arrow), and (**C**, **D**) hippocampal sclerosis on the left side in both anti-GAD65-positive patients (GAD65^+^, white arrows). **E** Neuronal cell loss is primarily observed in the CA1 (marked in the image with *) and CA3/4 (***) regions, whereas in subiculum (**) the neuronal populations appear well conserved in DBN- and GAD65-TLE (hematoxylin and eosin, upper panel). Dentate gyrus: marked by arrows. The extensive neurodegeneration is largely accompanied by extensive fibrillary astrogliosis, as evidenced by immunohistochemistry with antibodies against glial fibrillary acidic protein (GFAP; lower panel). Scale bars: overview: 500 μm; insets: 50 μm. (F) CD3⁺ and CD8⁺ T lymphocytes are present parenchymal and perivascular, partially located in apposition to neurons (arrowheads). These pathological features are accompanied by cellular infiltrates with features of microglia (CD68⁺) in CA4. Scale bars: 50 μm; insets: 10 μm. # and ##: location marked in GFAP overview

Cerebral magnetic resonance imaging (cMRI) scans of DBN-TLE patients showed swelling of the amygdala or severe loss of internal architecture of the hippocampus (Fig. 1A-C), which may be bilateral or unilateral. In DBN-TLE patients, a significant proportion (5/21) had HS and three patients had undergone epilepsy surgery (see Table 1 for clinical details). Anti-GAD65-AB-associated encephalitis also correlated with pathological MRI findings of temporal lobe involvement [17, 18] and parenchymal atrophy, with a subset of patients showing cortical/subcortical parenchymal T2 hyperintensity [19]. In total, 6/34 GAD65-TLE patients showed HS and three underwent surgical treatment for seizure relief. In summary, cMRI showed no neuroradiological differences between the two entities in terms of severe structural tissue damage involving the temporal lobes (Fisher’s exact test: 0.4116; for clinical details see Table 1).

**Table 1:** Clinical characteristics of GAD- and DBN-TLE patients

	GAD65+ (*n* = 35)	DBN+ (*n* = 22)	*p* value*	Odds Ratios	Confidence Interval (CI)
Age at onset (y), median (range)	30 (8–60)	51 (16–71)	0.0006		
Follow-up time (y), median (range)	9 (1–27)	4 (0–38)	0.0658		
Female, *n* (%)	23 (66)	9 (41)	0.0590		
Seizure outcome in latest follow-up					
Not seizure-free	25 (78)	9 (43)	0.0101	4.76	1.42–15.8
Seizure-free^c^	7 (22)	12 (57)			
Anti-seizure medication (ASM), *n* (%)	35 (100)	22 (100)			
Pharmacoresistance (yes), *n* (%)	24 (80)	8 (42)	0.0081	5.50	1.53–19.71
Pharmacoresistance (no), *n* (%)	6 (20)	11 (58)			
Immunotherapy (IT), *n* (%)^a^	23^a^ (66)	6^b^ (27)	0.0050		
Number of IT median (range)	2 (1–6)	1 (1–2)			
Not seizure-free after IT, *n* (%)	19 (90)	3 (60)	0.1548	6.33	0.63–63.62
Seizure-free after IT, *n* (%)^d^	2 (10)	2 (40)			
Hippocampal sclerosis (HS)			0.4116	0.59	0.11–3.24
HS yes, *n* (%)	6 (18)	5 (24)			
HS no, *n* (%)	28 (82)	16 (76)			
Epilepsy surgery	3 (9)	3 (14)	0.4252		
Surgery outcome^x^ Engel I, *n*	1	1			
Engel II, *n*	1	0			
Engel III, *n*	1	1			
Engel IV, *n*	0	1			

Odds Ratio calculator; Version 23.4.8, accessed February 3, 2026

1. Wieser HG, Blume WT, Fish D, et al. ILAE Commission Report. Proposal for a new classification of outcome with respect to epileptic seizures following epilepsy surgery. *Epilepsia*. Feb 2001;42(2):282-6

^a^Prednisolone (*n* = 20), Azathioprin (*n* = 4), IVIG (*n* = 5), Basiliximab (*n* = 4), Rituximab (*n* = 2), Mycophenolat Mofetil (*n* = 3), Immunadsorption (*n* = 3), PLEX (*n* = 2), Cyclophosphamid (*n* = 1)

^b^Prednisolone (*n* = 6), Rituximab (*n* = 1)

^c^seizure freedom at least for ≥ 6 mth independent of received therapy

^d^ seizure-free at 9–15 mth time point after IT start

^x^ Classification according to Engel ^1^

*Statistical comparisons between GAD- and DBN-TLE patients were performed using Fisher’s exact test for comparing the two categorical variables. Mann-Whitney U-test was used for nonparametric statistics

### HS and T cell infiltrates - neuropathological hallmarks of anti-DBN- and anti-GAD65-positive hippocampal biopsies

At the cellular level, in both DBN- and GAD65-TLE patients, marked segmental neuronal loss in CA1/CA3/4 and granular cell dispersion in the dentate gyrus (H&E, Fig. 1E) is accompanied by extensive reactive astrogliosis pronounced in segments with extensive neurodegeneration (GFAP, Fig. 1E, Suppl. Fig S1A). Additionally, hippocampi of DBN- and GAD65-TLE patients showed abundant innate and adaptive immune cell infiltrates including many macrophages, activated microglia (CD68, Iba1) and CTLs (CD3, CD8) in parenchymal and perivascular localization (Fig. 1F, Suppl. Fig. S1B). The near absence of granzyme B as a marker for cytotoxic effector T cells (Suppl. Fig S1C) is consistent with the protracted clinical course of the patients of whom hippocampal samples were available for analysis. 

### Single-nucleus RNA sequencing-based cell subtype cluster identification in brain biopsies from pharmacoresistant DBN- and GAD65-TLE patients

To learn in detail about shared or differential molecular and cellular pathomechanisms, unique cryopreserved biopsies of TLE patients positive for anti-DBN (*n* = 2) and for anti-GAD65 (*n* = 2) were used. As controls, samples from seronegative epilepsy patients were included, i.e. two patients with lesion-associated (LA) epilepsy, one patient with dysembryoplastic neuroepithelial tumor, and one with cortical dysplasia both not involving hippocampal structures, and two patients with classic HS without a history of inflammatory episodes or AE (see Table 2 for detailed clinical data).

**Table 2:** Clinical characteristics of GAD65- and DBN-TLE patients and controls used for snRNA-seq. Study group LA, HS controls. Study group GAD65-/DBN-TLE patients

	*LA1*	*LA2*	*HS1*	*HS2*
sex	m	m	f	f
age at onset (years)	0.75	4.5	0.2	6
age at surgery (years)	28.7	59.8	11.1	57.7
time onset to surgery (years)	28.0	55.3	11.0	51.7
MRI (pre-surgery)	na	suspected hippocampal sclerosis (left), Cystic lesion temporomesial with displacement of the hippocampus (left)	hippocampal sclerosis (left), suspicion of abnormal cortico-medullary differentiation in the temporal pole (left)	hippocampal sclerosis (right)
last therapeutic regimen pre-surgery • ASM	LTG, TPM, CMZ	LCM, ESL	LEV, OXC	LTG, ZNS
• IT	-	-	-	-
surgery	amygdala-hippocampectomy and resection frontal operculum, insular cortex (right)	Amygdala-hippocampectomy and epidermoid resection (left)	amygdala-hippocampectomy and temporal pole resection (left)	amygdala-hippocampectomy and resection of the piriform cortex (right)
seizure severity (pre-surgery)	FIAS: daily; generalized: <1/year	FAS 2/month; FIAS: 2/month	FAS: 4/week; FIAS: 2-3/month	FAS: every 6 weeks; FIAS: 5/month; FBTC: <1/year
outcome surgery^a^	Engel IB	na	Engel IIIA	Engel IA

	*GAD1*	*GAD2*	*DBN1*	*DBN2*
sex	f	f	m	f
age at latest FU (years)	53.8	55.9	27.1	55.5
age at onset (years)	31	43	21	15
age at surgery (years)	53	51	25.6	38.4
time onset to surgery (years)	22.6	8.8	4.6	23.4
MRI (pre-surgery)	hippocampal sclerosis and signal increase of the amygdala (left), discrete signal increase amygdala (right)	hippocampal sclerosis (left), signal increase amygdala (left), weak signal increase hippocampal formation (right)	hippocampal sclerosis (left)	hippocampal sclerosis (right), hemiatrophy (right)
treatment history (pre-surgery) • ASM	VPA, ZNS, LTG, TPM, PB, LCM, LEV, CMZ	LEV, OXC	LEV, OXC	VPA, LTG, LCM
• IT	-	steroid pulse	-	-
last therapeutic regimen before surgery • ASM	LEV, CMZ	LEV, OXC	LEV, OXC	LTG, LCM
• IT	-	steroid pulse (last pulse 0.5 years before surgery)	-	-
surgery	amygdala-hippocampectomy (left)	amygdala-hippocampectomy (left)	amygdala-hippocampectomy (left)	amygdala-hippocampectomy and anterior temporal lobe recection (right)
seizure severity (pre-surgery)	FIAS: daily	FIAS: 20/month; PNES: <1/month	FIAS: 1-2/week	FIAS: 2-3/month
outcome surgery^a^	Engel IIIA	Engel IID	Engel IA	Engel IV

*Abbreviations*: *ASM* anti-seizure medication, *CMZ* carbamazepine, *ESL* eslicarbazepine acetate, *FAS* focal aware seizure, *FBTC* focal to bilateral tonic-clonic seizure, *FIAS* focal impaired awareness seizure, *HS* hippocampal sclerosis, *IT* immunotherapy, *LA* lesion-associated, *LCM* lacosamide, *LEV* levetiracetam, *LTG* lamotrigine, *OXC* oxcarbazepine, *TPM* topiramate, *ZNS* zonisamide

^a^Classification according to Engel (Wieser et al., ILAE Commission Report. Proposal for a new classification of outcome with respect to epileptic seizures following epilepsy surgery. Epilepsia. Feb 2001;42(2):282-6.)

**Fig. 2:**
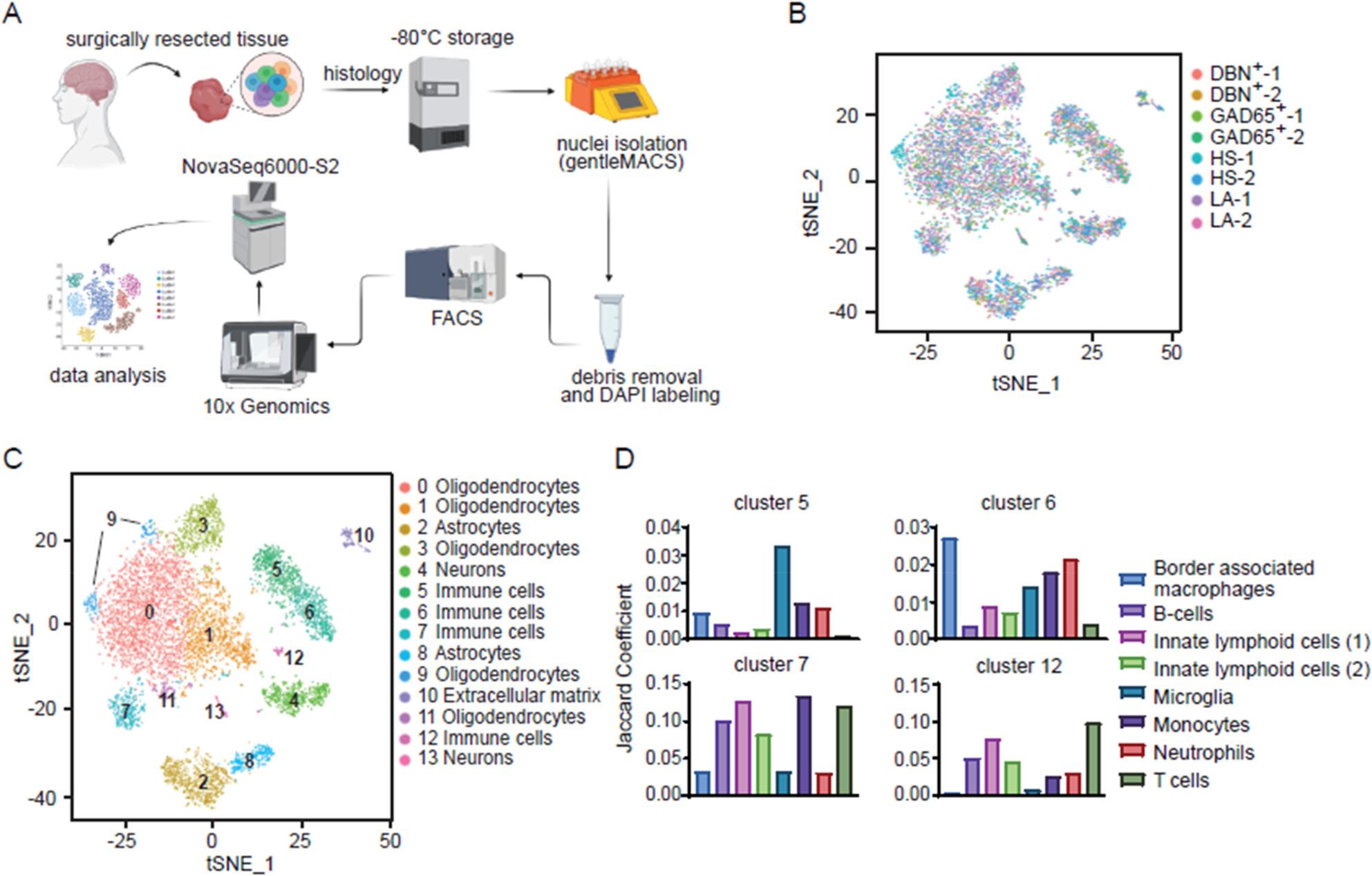
Pronounced adaptive immune cell transcript signatures in hippocampal biopsies of GAD65- and DBN-TLE patients versus controls. **A** Schematic representation of the snRNA-seq method on fresh-frozen hippocampal tissue with nuclei sorted based on DAPI staining. **B** t-distributed stochastic neighbour embedding (tSNE) plot of all recovered nuclei from the eight individual patients. **C** tSNE plot combined with cellular clustering and classification revealed 14 individual clusters with distinct transcriptional profiles. **D** The Jaccard Coefficient of each immune clusters (i.e. clusters 5, 6, 7 and 12) based on single cell cite-seq data of Golomb et al. [20]. BAM= Border associated macrophages. Created with BioRender.com

We investigated the transcriptional profiles of the four clinical subgroups (anti-GAD65, anti-DBN, LA, HS), using snRNA-seq from fresh-frozen hippocampal tissue with nuclei sorted based on DAPI staining (Fig. 2A). In total, 8011 nuclei were sequenced that passed quality control and doublet filtering (see Materials and Methods for quality control parameters). Sex-affected genes were excluded and all nuclei were integrated using Principal Component Analysis (PCA), Uniform Manifold Approximation and Projection (UMAP) and t-distributed Stochastic Neighbour Embedding (tSNE) clustering (Fig. 2B, Suppl. Fig. S2A). The individual samples showed a high-level integration with no discrete sample-specific clusters, except for a small population of nuclei derived from one patient (HS-1) (Fig. 2B). Subsequent cellular clustering with clustering resolution of 0.4, and classification revealed 14 individual clusters with distinct transcriptional profiles within the tSNE plot (Fig. 2C, Suppl. Fig. S2B-D, S3). A combinatorial approach of GO annotation and snRNA-seq marker sets (see Materials and Methods) confirmed the major cell types of the corresponding clusters: i.e., clusters 0, 1, 3, 9 and 11 represent oligodendrocytes; clusters 2 and 7 represent astrocytes; clusters 5, 6, 7 and 12 were annotated as immune cell-enriched populations, and clusters 4 and 13 represent neurons (Fig. 2C).

### Adaptive and innate immune cell abundance in hippocampi of patients with GAD65- and DBN-TLE

Key clusters were defined by immune cell types (Fig. 2C, clusters 5, 6, 7 and 12) based on immune single cell cite-seq data from Golomb et al. [20]. The Jaccard Coefficient of each immune cluster revealed that the nuclei present in cluster 5 are mainly microglia and in cluster 12 are mainly T cells. Further two immune clusters (clusters 6 and 7) consisted of a more heterogeneous population of immune cells, both containing microglia, border-associated macrophages (BAM), innate lymphoid cells, monocytes, neutrophils, and B and T cells (Fig. 2D). To more directly assess the identity of microglia within these clusters, we examined single-gene expression patterns rather than relying solely on the Jaccard Coefficient. Clusters 5 and 6 expressed canonical homeostatic microglial markers (P2RY12, TMEM119, CX3CR1) and TGFB1, with minimal CCR2 or S100A8/A9 expression. HLA-DRA (MHC-II) was detected, consistent with activation of resident microglia rather than infiltration by peripheral monocytes. These findings are consistent with clusters 5 and 6 representing resident activated microglia, whereas cluster 7 lacked a clear monocyte signature (Suppl. Fig. S4, Suppl. Table 1). Importantly, canonical microglial activation markers, including APOE, SPP1, CST3, and CD74, were readily detected across microglial clusters (Suppl. Table 1), and were present in all four clinical subgroups. Further subclustering of clusters 5, 6, 7 and 12 did not improve interpretability, as nuclei redistributed by age and sex, whereas our original SCTransform-based integration (see Methods) maintained a more balanced distribution across samples (Suppl. Fig. S5).

Intriguingly, the hippocampi of the two AB-positive groups had a higher proportion of immune cells compared to the non-AB control samples (Suppl. Fig. S2E, left panel). In addition, more T cells were found in the hippocampi of the GAD65- and DBN-TLE patients compared to the non-AB samples (Suppl. Fig. S2E, right panel), which was accompanied by a higher expression level of CD8a and CD8b in these samples (Suppl. Fig. S6, Suppl. Table 1). No differences were observed between the AB-positive vs. non-AB controls for the immune markers CD4 and CD3e, the cytotoxic effector genes GZMB and PRF1, and exhaustion-associated markers PDCD1 and CTLA4. Furthermore, IFN-γ (IFNG) expression was nearly absent across all samples, suggesting that strong interferon signaling is not a prominent detectable feature in this human hippocampal snRNA-seq datasets (Suppl. Fig. S6, Suppl. Table 1).

### Immune pathway related transcript signatures in patients with GAD65- and DBN-TLE

**Fig. 3:**
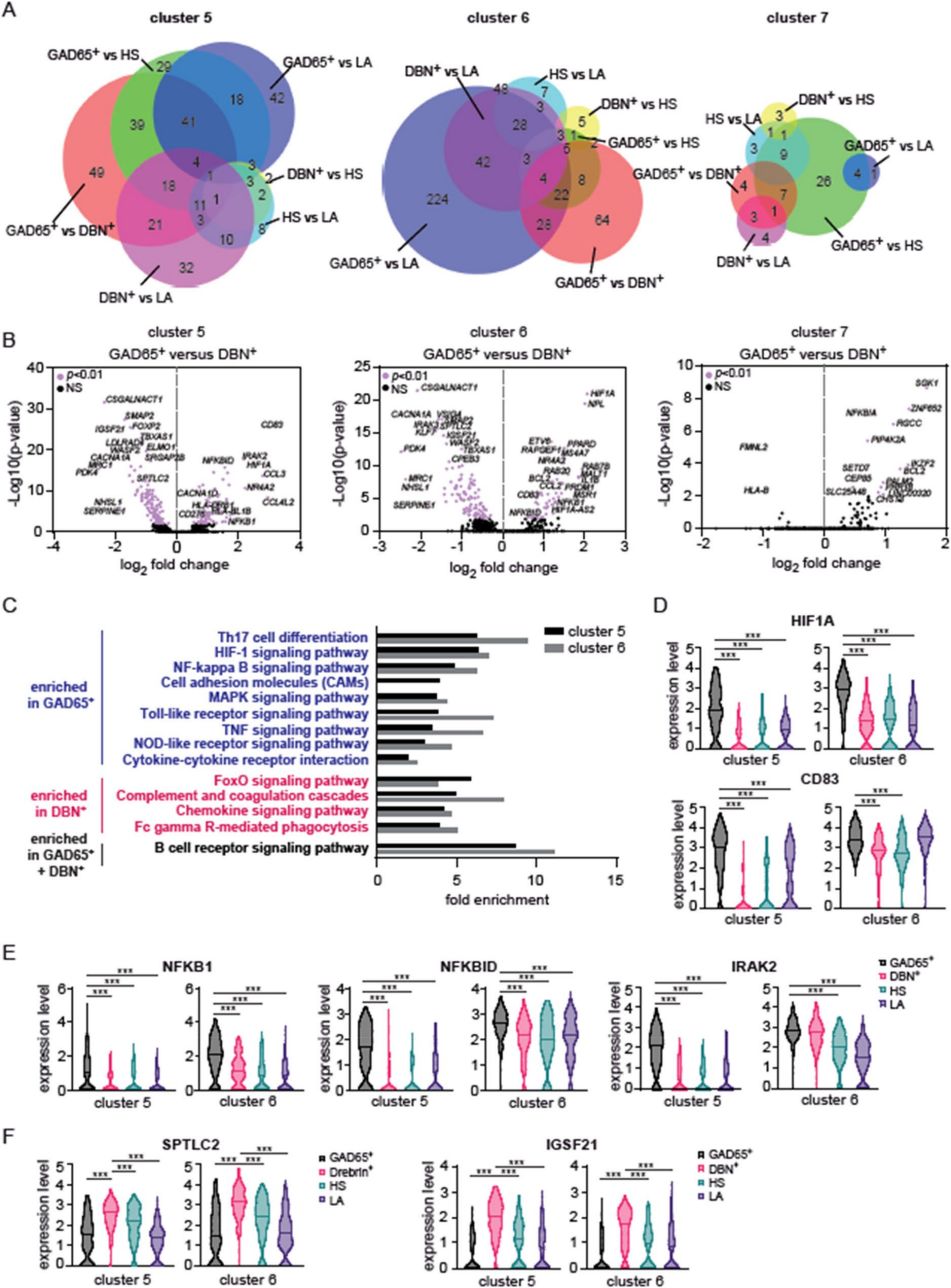
Anti-GAD65 and anti-DBN hippocampal snRNA-seq signatures indicate differential immune pathway signaling. **A** DeepVenn diagrams of the differentially expressed genes between the four clinical subgroups of cluster 5 (left panel), cluster 6 (middle panel) and cluster 7 (right panel). The numbers represent the number of genes differentially expressed between the different groups. **B** Volcano plots between GAD65- and DBN-TLE patients in the immune clusters 5, 6 and 7 (*p*-adj < 0.01, NS = not significant). **C** GO-analysis of differentially expressed genes (*p*-adj < 0.01) between anti-GAD65- and DBN-TLE patients in clusters 5 and 6. **D**-**F** Expression levels for individual genes, differentially expressed in hippocampi of GAD65-**D**, **E** or DBN-TLE (**F**) patients (***p < 0.001). GAD65^+^ = anti-GAD65-positive TLE, DBN^ +^ = anti-DBN-positive TLE, HS = hippocampus sclerosis, LA = lesion-associated patients

Next, we compared the snRNA-seq transcriptomes of the four different clinical subtypes in the three major immune clusters (clusters 5, 6 and 7). Cluster 12 was excluded from further analyses due to too few nuclei. DeepVenn analysis [21] of the differentially expressed genes (DEGs) between the four clinical subtypes revealed fundamentally distinct expression signatures (Fig. 3A). From cluster 5, 49 genes were differentially expressed between GAD65- and DBN-TLE patients, but these genes were not affected in other clinical subtypes (Fig. 3A, left panel). A similar scenario was evident for cluster 6, whereas for the smaller cluster 7 only a few genes were subtype specific (Fig. 3A, middle and right panels).

We next focused on transcriptomic differences between the two AB-positive subtypes to identify GAD65- and DBN-TLE patient-specific immunological profiles. Volcano plots for clusters 5, 6 and 7 revealed distinct transcriptional profiles of the two subtypes (Fig. 3B). For cluster 5, a total of 214 genes were differentially expressed (*p*-adj < 0.01), with 82 genes augmented in GAD65-TLE and 132 genes increased in DBN-TLE patients. For cluster 6, 163 genes were differentially expressed (*p*-adj < 0.01), with 56 genes increased in GAD65-TLE and 107 genes in DBN-TLE, and for cluster 7, 16 genes were differentially expressed (*p*-adj < 0.01), with 2 genes induced in GAD65-TLE and 14 genes in DBN-TLE (Fig. 3B, Suppl. Table 2). Given that only 16 genes were differentially expressed in cluster 7, of which four were specific to GAD65- or DBN-TLE patients (i.e. PALM2, CHST3, PRR18 and FMNL2), we next focused on clusters 5 and 6 for further analyses.

GO-analysis was performed on DEGs between GAD65- and DBN-TLE. In the hippocampi of the anti-GAD65-positive patients, DEGs from clusters 5 and 6 were associated with several immunological pathways, including the NF-κB pathway, Tumor necrosis factor (TNF), Nucleotide-binding and oligomerization domain (NOD)-like receptor, Hypoxia-inducible factor-1 (HIF-1), Toll-like receptor signaling pathways and Th17 cell differentiation. In contrast, we observed enrichment for Forkhead box O (FoxO) and chemokine signaling pathways, complement and coagulation cascades, and Fc gamma R-mediated phagocytosis in anti-DBN-positive patients. The B cell receptor signaling pathway was also enriched in both patient groups (Fig. 3C).

Analysis of transcriptional profiles in clusters 5 and 6 revealed distinct GAD65-TLE expression profiles for a subset of genes, including HIF-1 A, CD83, and several components of the NF-κB signaling pathway (NFKB1, NFKBID, IRAK2) (Fig. 3D, E), consistent with enrichment of the HIF-1 and NF-κB signaling pathways observed in the GO analysis. Specific expression profiles were additionally observed in the hippocampi of DBN-TLE patients, as visualized by the significant increase in expression of SPTLC2 and IGSF21 (Fig. 3F).

### Differential transcription factor enrichment in hippocampal mRNA of GAD65- versus DBN-TLE patients

**Fig. 4:**
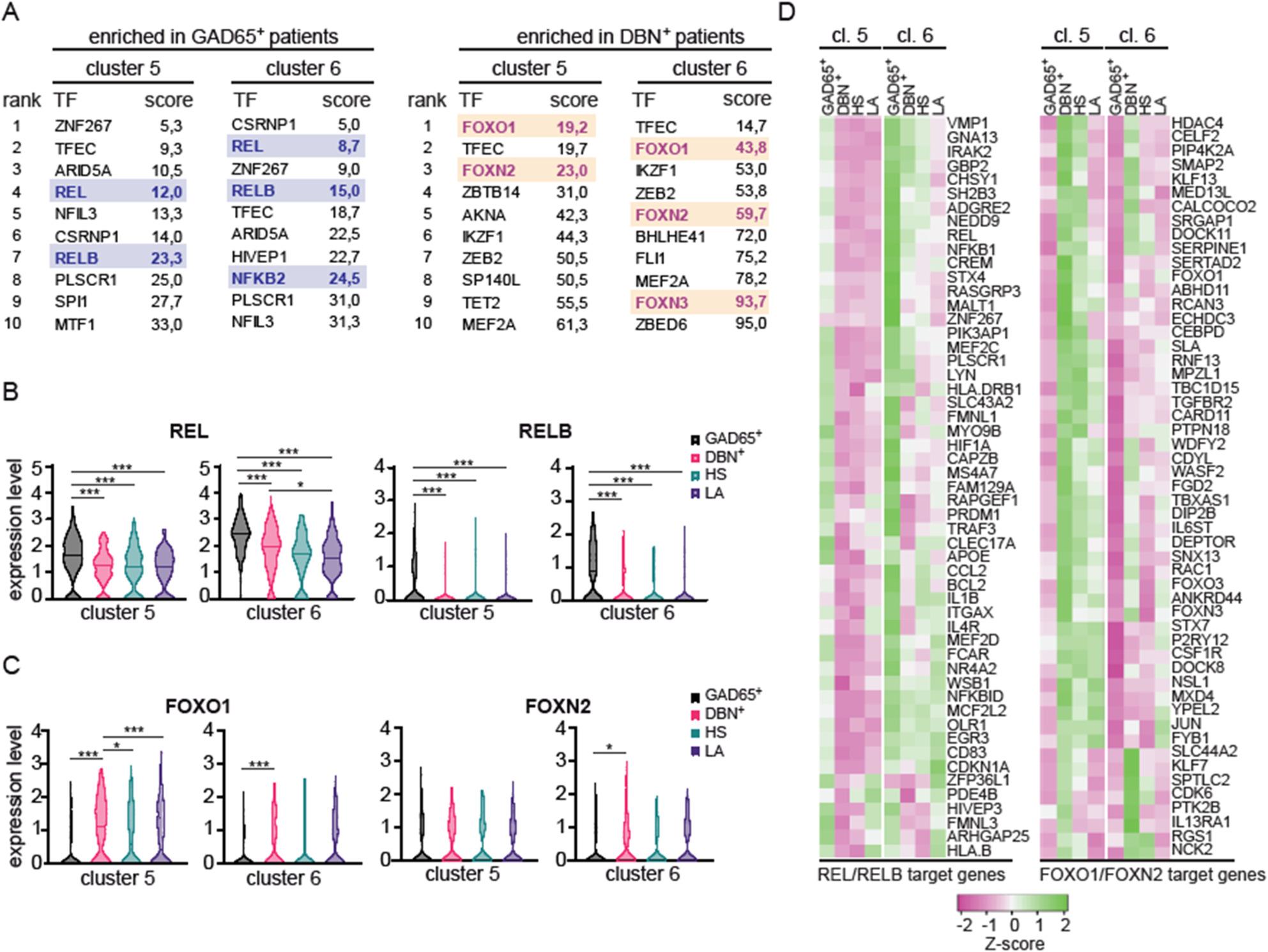
Distinct master regulators and dependent transcript sets in GAD65- versus DBN-TLE hippocampi. **A** Transcription factor enrichment of the differentially expressed genes of the clinical subgroups GAD65- (left panel) and DBN-TLE (right panel) in clusters 5 and 6. The ten transcription factors with the highest score (based on ChEA 2022, ChEA Encode consensus, TTRUST 2019, JASPAR and TRANSFAC and Protein-Protein interaction) are given, with members of the NF-κB pathway given in blue, and members of the FOX family shown in pink (**B**, **C**) Expression levels for individual transcription factors belonging to the NF-κB pathway (**B**) and belonging to the FOX-related transcripts (**C**) (*p < 0.001, ***p < 0.001). **D** Heat map of target genes for the transcription factors REL and RELB (left panel) and the transcription factors FOXO1 and FOXN2 (right panel) in clusters 5 and 6 for the four clinical subgroups

To investigate if GAD65- and DBN-TLE-specific transcriptional profiles might be controlled by one or more transcription factors (TFs), suggestive of AB-specific regulation, we performed TF enrichment analysis (TFEA) on GAD65- and DBN-TLE-specific DEGs in clusters 5 and 6. TFEA prioritizes TFs based on the overlap between the DEGs and annotated TF targets [22]. Interestingly, for GAD65-TLE-specific DEGs, enrichment for several TFs belonging to the NF-κB signaling pathways, i.e. REL, RELB and NFKB2, was observed for both clusters (Fig. 4A, left panel). In contrast, TFs belonging to the Fox family (FOXO1, FOXN2 and FOXN3) were clearly enriched for DBN-TLE-specific DEGs (Fig. 4A, right panel).

Expression analysis of the top-ranked TFs of the NF-κB- and Fox-related transcripts in the four subgroups revealed a GAD65-TLE-specific increase for the TFs REL and RELB and an anti-DBN-specific abundance for the TFs FOXO1 and FOXN2 in the two largest immune clusters (Fig. 4B, C). Consistently, per-cell-type module scoring showed that NF-κB– and FoxO-associated signatures were most prominent in microglial-enriched clusters (clusters 5 and 6), whereas cluster 7 displayed low activity for both pathways (Suppl. Fig. S7A). Anti-GAD65-positive samples clustered closely together for both signatures in cluster 5, and both anti-GAD65-positive and anti-DBN-positive samples forming distinct groupings in cluster 6. Notably, HS and LA control samples partially overlapped with the anti-DBN group in cluster 5, whereas in cluster 6 they displayed a more intermediate distribution, not clearly aligning with either autoimmune group. Moreover, immunohistochemical co-staining with neuronal and astrocytic markers showed NF-κB- and FOXO-associated proteins in both neuronal and glial compartments of the hippocampal tissue (Suppl. Figure 7B). Importantly, the specific enrichment of NF-κB– and FoxO-associated programs represents a transcriptional endophenotype derived from exploratory snRNA-seq analyses and was not captured by conventional histopathological assessment or protein-level quantification.

We then explored the expression of genes targeted by REL and RELB as well as for the TFs FOXO1 and FOXN2, i.e. genes with at least one annotated binding site for REL/RELB or FOXO1/FOXN2. A heat map of the expression profiles of the target genes in clusters 5 and 6 revealed clear segregation of REL/RELB target genes for the GAD65-TLE group (Fig. 4D, left panel) and a distinct pattern for FOXO1/FOXN2 target genes in the DBN-TLE group (Fig. 4D, right panel), consistent with differential NF-κB- and FoxO-associated signaling in GAD65- and DBN-TLE patients, respectively.

To verify that the observed NF-κB- and Fox-related transcriptional signatures were not driven by limited transcript detection, we assessed the abundance of the genes included in the REL/RELB and FOXO1/FOXN2 target gene analyses across the immune clusters. Aggregated read counts for these gene sets ranged from approximately 10,000 to 60,000 reads per cluster, with the strongest signal observed in the larger microglial-enriched clusters 5 and 6 (Suppl. Figure 8 A). Across individual samples, most genes were consistently detected with approximately 100–200 reads per cluster, while several genes exceeded 1,000 reads per cluster. At the single-cell level, median raw counts exceeded 50 reads per gene across the major immune clusters (Suppl. Figure 8B). These findings indicate sufficient detection of REL/RELB- and FOXO1/FOXN2-associated transcripts in the immune compartment and support the biological interpretation of the observed transcriptional differences.

### Distinct therapy responses between DBN- and GAD65-TLE patients

Epilepsy with ABs against the intracellular protein GAD65 is known to be pharmacoresistant to anti-seizure medication (ASM) and adequate immunomodulatory treatment (IT) regimens remain a clinical challenge [23, 24]. Accordingly, in the present GAD65-TLE cohort, 80% (24/35) were pharmacoresistant defined as failure of sustained seizure freedom after adequate trials of two tolerated, appropriately selected and used ASM regimens according to the ILAE definition [25]. By contrast, in DBN-TLE patients, only 42% (8/22) were pharmacoresistant to ASM (Table 1; Fisher’s exact test: *p* = 0.0081). Under IT, only 10% (2/23) of GAD65-TLE patients were seizure free within a 9-month interval, with Basiliximab (*n* = 1), or prednisolone (*n* = 1). Two anti-DBN-positive patients improved with IT receiving prednisolone (*n* = 1), or rituximab (*n* = 1). Strikingly, significantly fewer patients in the DBN-TLE cohort received IT (Fisher’s exact test: *p* = 0.005; Table 1). Moreover, GAD65-TLE patients were exposed to a broader range of immunotherapeutic interventions. These differences in treatment exposure may represent a potential confounding factor in treatment-response analyses and should therefore be interpreted with caution (see Table 1 for details).

### Distinct pharmaco-response potentials indicated by anti-GAD65 and anti-DBN hippocampal RNA signatures

To explore potential therapeutic targets associated with pathways and genes differentially expressed between the hippocampi of DBN- and GAD65-TLE patients, we performed exploratory drug–gene interaction analyses. The DGIdb database (https://dgidb.org) ^27^ was used to identify compounds predicted to interact with components of the FoxO and NF-κB pathways. Several pharmacological compounds were predicted to interact with proteins encoded by FOXO (FOXO1, FOXO3) and NF-κB transcripts (NFKB1, NFKB2), including drugs with approved regulatory status. Notably, antioxidant compounds such as resveratrol were predicted to interact with NFKB2, NFKB1 and FOXO3 (Suppl. Fig. S9A).

Subsequently, genes within the REL/RELB and FOXO1/FOXN2 networks were analysed using the LINCS L1000 resource (https://maayanlab.cloud/Enrichr/), and DGIdb databases to identify perturbagens associated with these transcriptional signatures. Within this framework, compounds such as prednisone were predicted to decrease expression of the REL/RELB target genes, whereas perturbagens linked to the FOXO1/FOXN2 network were predicted to increase expression of their target genes, including immunosuppressive agents such as dexamethasone (Suppl. Fig. S9B).

Finally, using the L1000CDS² search engine (https://maayanlab.cloud/L1000CDS2), we identified small molecules computationally predicted to counteract aspects of the transcriptomic signature of DEGs in GAD–TLE patients. Cluster 5 DEGs (Figs. 2B and 3B), associated with altered microglial gene expression, were used as input. L1000CDS² leverages the LINCS L1000 resource, which profiles transcriptional responses of four human cancer cell lines to ~ 1,300 drugs for 6 h. Top ranked compounds included immunosuppressive agents such as loteprednol, fluticasone, and dexamethasone (Suppl. Fig. S9C), which were predicted to modulate genes associated with Fox-related signaling pathways (Suppl. Fig. S9D). Together, these analyses provide a hypothesis-generating framework linking disease-associated transcriptional signatures to potential pharmacological perturbations. These analyses are purely computational and do not imply therapeutic efficacy or clinical applicability.

## mRNA signatures of a T cell-driven mouse model of limbic encephalitis

Considering the immune cell infiltrates observed in human hippocampal biopsies, we next aimed to identify key transcription factors and pathways in a mouse model of LE with a fully defined pathogenetic sequence. To this end, we used the OVA-CD8⁺ LE model, a highly cytotoxic system in which antigen-specific CD8⁺ T cells induce hippocampal inflammation. This model captures selected features of human CTL-mediated encephalitis, including seizures, hippocampal sclerosis, and immune cell infiltration [26]. In these mice, OVA is selectively expressed in hippocampal neurons of transgenic OT-I/Rag1⁻/⁻ mice harboring OVA-specific CD8⁺ T cells.

**Fig. 5:**
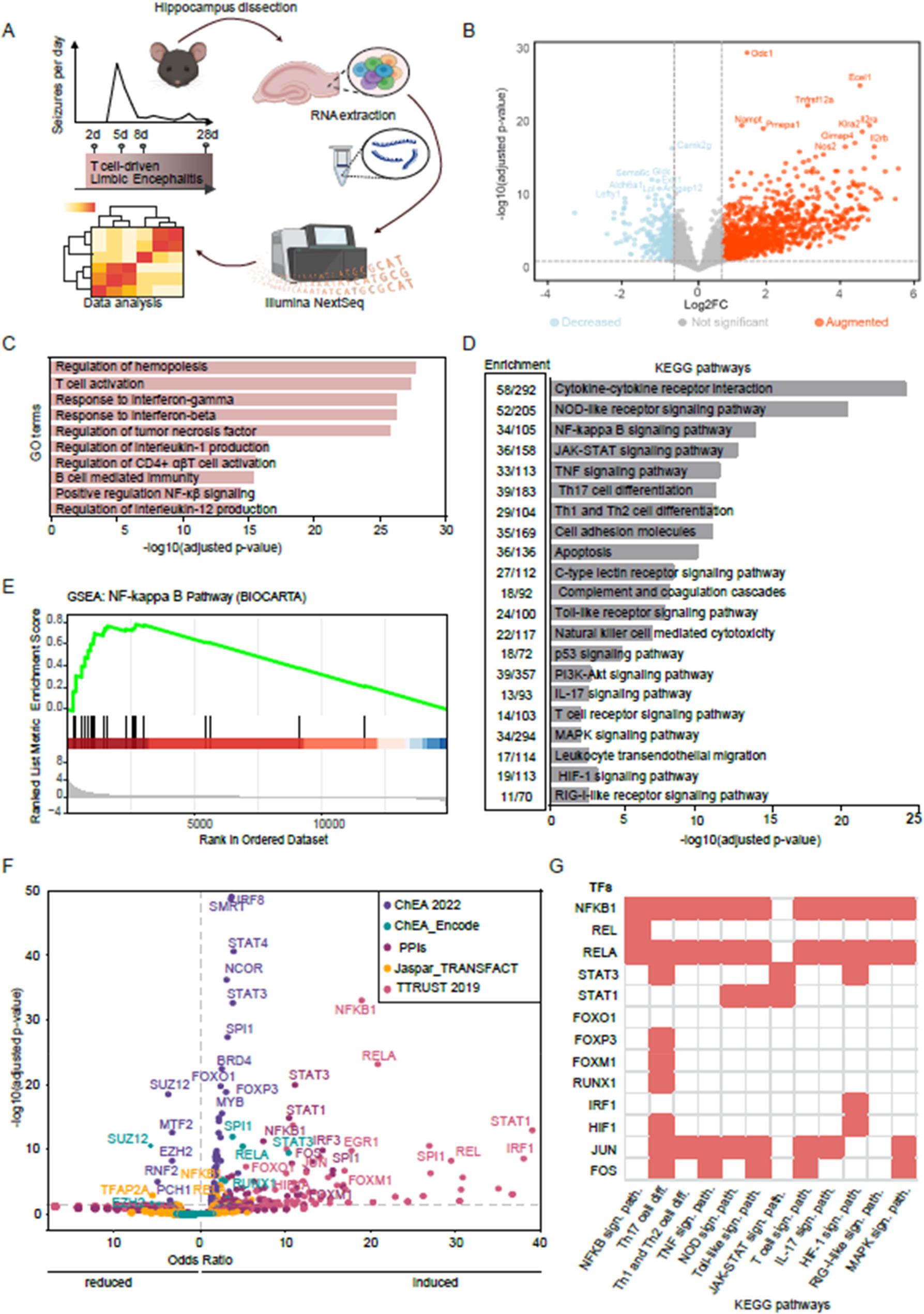
NF-κB pathway regulated RNA signatures emerge from acute T cell-driven experimental limbic encephalitis. **A** Schematic representation of bulk RNA-seq analysis conducted from different time points in (2d, 5d, 8d, and 28d) hippocampi of a T cell-driven limbic encephalitis mouse model (OVA-CD8^+^ LE mice). **B** Volcano plot visualize correlation of statistical significance (Adj. *p* value) versus magnitude of change (Log2FC) for differentially expressed genes (DEG) between rAAV-OVA-injected and corresponding controls at 5 days after injection (FDR ≤ 0.05). The direction is defined by Log2FC; for the induced genes Log2FC ≥ 0.66 (red) and for the reduced ones Log2FC ≤ -0.66 (blue) as well as variable direction within the comparisons (grey). **C** Most significant GO terms of the corresponding increased (red) and decreased (blue) DEGs. **D** KEGG pathway analysis (upper panel) of increased DEGs (Log2FC ≥ 0.66, FDR ≤ 0.05) categorized by their Enrichment (DEGs for the pathway /Pathway-associated genes) and Adj. *p*-value. **E** GSEA analysis (lower panel) of the pathway with higher enrichment. **F** Transcription factor enrichment (TFEA) from distinct databases (ChEA 2022, ChEA Encode consensus, TTRUST 2019, JASPAR and TRANSFAC and Protein-Protein interaction) of the DEGs (Log2FC ≥ I0.66I, FDR ≤ 0.05). **G** Matrix correlating transcription factors found in the TFEA analysis and the 20 most relevant KEGG pathways enriched by the increased genes (Log2FC ≥ 0.66, FDR ≤ 0.05). Seizure pattern representation derived from Pitsch et al., [26]. Created with BioRender.com

To assess transcriptional changes, we compared mRNA expression profiles in hippocampi at multiple time points representing distinct stages of disease progression (days 2, 5, 8, and 28) following injection with rAAVs encoding the OVA gene under a neuron-specific promoter (rAAV-hSyn-OVA-mCherry; rAAV-OVA group) or a control vector expressing only a fluorescent marker (rAAV-hSyn-mCherry; rAAV-control group; Fig. 5A). We identified two distinct clusters in the PCA. The first cluster comprised all samples from the rAAV-control group across all time points, as well as the rAAV-OVA samples at day 2. The second cluster included rAAV-OVA samples at days 5, 8, and 28 (Suppl. Fig. S10A). Comparative transcriptomic analyses across disease stages revealed prominent transcriptional alterations at multiple time points. The majority of differentially expressed genes (DEGs) were detected at 5 days post vector-based antigen transfer (1426), followed by 8 days (1242) and 28 days (1092), whereas only minimal changes were observed at 2 days (5) (Log2FC ≥|0.66|, FDR ≤ 0.05) (Suppl. Table 3). Despite representing distinct disease stages, a substantial overlap in DEGs across time points was observed (Suppl. Fig. S10B).

For comparison of overlapping inflammatory programs with human GAD– and DBN-TLE, the five-day time point was selected to capture the previously described seizure-prone period around days 4–7, corresponding to an acute phase of robust hippocampal inflammation [26]. This acute inflammatory stage at day 5 was characterized primarily by increased gene expression, with 1,132 DEGs upregulated versus 295 DEGs downregulated (Fig. 5B). Functional GO analysis of the upregulated DEGs was then performed to identify inflammatory cascades. Interestingly, the increased DEGs revealed altered pathways related to processes of the adaptive (T cell activation) and innate (response to interferon-gamma and -beta) immune system (Fig. 5C).

Subsequent KEGG analysis of the upregulated DEGs supported transcript enrichment related to both innate and adaptive immune responses with the greatest overlap observed with human anti-GAD65–positive TLE. At five days post-antigen transfer, numerous inflammatory processes were significantly enriched, including the NF-κB, TNF, NOD-like receptor, and Toll-like receptor signaling pathways, as well as Th1, Th2, and Th17 cell differentiation (Fig. 5D). The enrichment of transcripts for these inflammatory pathways was also observed in later time points, suggesting sustained activation of immune-related transcriptional programs over time that may reflect cytotoxic dynamics (Suppl. Fig. S10C). Notably, NF-κB–associated DEGs overlapped across time points, indicating that many of these genes remain differentially expressed throughout disease progression (Suppl. Fig. S10D).

Furthermore, Gene Set Enrichment Analysis (GSEA) of the NF-κB signaling pathway (Biocarta database) revealed significant enrichment among differentially expressed genes at day 5 (Fig. 5E), indicating an overexpression of NF-κB-associated transcriptional signatures within the observed inflammatory gene expression profile. Finally, annotation of these augmented DEGs with immune cell types, based on single-cell RNA-seq data from Golomb et al. [20] revealed a highly heterogeneous population enriched for gene signatures associated with microglia and monocytes (Suppl. Fig. S10E).

To unravel the transcriptional mechanisms associated with the above-described functional GO terms, TFEA was performed from different databases (ChEA 2022, ChEA Encode consensus, TTRUST 2019, JASPAR and TRANSFAC and Protein-Protein interaction). Our TFEA analysis revealed that many TFs were significantly enriched, especially for the augmented DEGs. For example, the STAT TFs, represented by several family members (STAT1-4), but also the immediate early TFs (e.g. EGR1, JUN), TFs of the HIF1 (HIF1A) and NF-κB (NFKB1, REL and RELA) were strongly enriched (Fig. 5F). In addition, several Fox genes (FOXO1, FOXP3, FOXM1) were found to be enriched (Fig. 5F). The relevance of these TFs to the activation of altered signaling pathways observed in the KEGG analysis (Fig. 5D) was also highlighted schematically (Fig. 5G). TFs linked to the NF-κB signaling pathway were prominently represented among regulators associated with KEGG pathways enriched for upregulated DEGs (Fig. 5G), further highlighting the association of NF-κB signaling with the inflammatory transcriptional program observed in the OVA-CD8^+^ LE mouse model.

## Discussion

The present study provides an exploratory characterization of rare hippocampal tissue from DBN- and GAD65-associated TLE and identifies broad similarities in neuropathological damage as well as CTL and microglial infiltration patterns. In contrast, snRNA-seq analyses reveal differences in microglia-associated transcriptional states between the two groups, suggesting that similar pathological endpoints may arise from partially distinct underlying inflammatory transcriptional programs.

CTL-associated immune activity emerges as a prominent feature in both DBN- and GAD65-TLE, and may contribute, particularly in early disease stages, to the observed pathology. This interpretation is consistent with findings from experimental CD8^+^ T cell–driven models such as the OVA-CD8^+^ LE mouse model [26]. In this model, seizure frequency decrease markedly following CTL-induced neuronal loss. In contrast, DBN- and GAD65-TLE patients exhibit heterogeneous and temporally dynamic seizure phenotypes over prolonged disease course. Clinical staging frameworks in anti-GAD65-positive TLE further support a transition from an early active inflammatory ‘encephalitic’ phase (≤ 6 years) to a later immunologically ‘low active’ phase (> 6 years), which correlates with CTL abundance in surgical tissue [3]. Similar stage-dependent immune dynamics have been observed in CD8⁺ T cell-driven disorders, such as Rasmussen’s encephalitis, where early lesions show pronounced CTL infiltration, whereas chronic stages are characterized by neuronal loss, synaptic remodeling, and reduced T cell presence [27]. In line with this, our cohort, characterized by long durations between onset and surgery (GAD65-TLE patients: onset at 31 and 43 years, with surgery 22.6 and 8.8 years later; DBN-TLE patients: onset at 21 and 15 years, with surgery 4.6 and 23.4 years later; Table 2) showed pronounced structural damage and relatively limited residual CTL presence. Consistent with prior studies, advanced structural damage and prolonged CTL activity have been associated with reduced responsiveness to immunotherapy despite evidence of T cell exhaustion [28]. One anti-GAD65-positive patient had received immunotherapy without clinical improvement. Although demographic and clinical differences, including age at onset, disease duration, and treatment exposure may act as confounders, all cases reflect long-standing, treatment-refractory disease stages with substantial structural pathology. These observations support a model in which CTLs contribute to early epileptogenic injury, whereas additional mechanisms likely sustain chronic inflammation and network dysfunction in later stages.

Within the immune compartment of resected hippocampal tissue, microglia constitute the predominant immune cell population as captured by snRNA-seq. Exploratory transcriptomic analyses identify differential enrichment of FoxO- and NF-κB–associated gene expression programs between disease groups. These signatures likely reflect cell-state–associated transcriptional programs in chronically affected tissue rather than functional pathway activation.

Several factors may contribute to the differences in FoxO or NF-κB signaling. These include variability in antigenic targets of T cell receptors, AB properties, and their effects on neuronal signaling pathways. CTL-associated acute neuronal injury involves perforin and granzyme-dependent cytotoxicity [29], contributing to neuroinflammation [30, 31]. In our samples, granzyme B staining was largely absent, consistent with a chronic disease rather than ongoing acute cytotoxicity. Nevertheless, earlier inflammatory phases may have established a persistent cytokine milieu that is capable of sustaining NF-κB signaling [32, 33]. Given limited tissue availability, not all histological assays could be uniformly performed across cases.

Host genetic factors may further modulate pathway activity. A recent genome-wide association study (GWAS) in anti-GAD65-positive patients implicate regulator, such as Protein Kinase C Beta (PRKCB) and Proto-oncogene tyrosine-protein kinase (SRC), which influence NF-κB signaling [34–36]. Similar genetic data are currently unavailable for DBN-TLE. In addition, the hippocampal microenvironment, including immune cytokines, immune cell composition, and inflammatory mediators, likely contributes to differential pathway states. Indeed, NF-κB and FoxO pathways exert partly opposing roles in inflammatory regulation [37–40]. FOXO3 has been proposed as a negative regulator of NF-κB signaling [41], while FoxO signaling in microglia may support stress resistance and cellular homeostasis [42, 43]. Distinct TF programs, such as FOXP1 and RBFOX1/2 have also been implicated in inflamed microglial states in disorders such as Rasmussen’s encephalitis [44].

In DBN-TLE, AB-associated mechanisms may indirectly shape microglial state and contribute to FoxO-associated transcriptional programs. This is consistent with AE, where ABs have been linked to neurodegenerative processes beyond their biomarker role [45]. In contrast, NF-κB signaling in microglia is associated with pro-inflammatory activation states, characterized by cytokine, chemokine, and reactive oxygen species (ROS) production [46], potentially contributing to chronic neuroinflammation in anti-GAD65-positive TLE. These divergent programs may relate to differences in neuronal integrity, synaptic plasticity, and clinical course, although direct causal inference is not possible.

Module scoring analyses indicate that FoxO and NF-κB signatures are enriched in distinct microglial subsets and vary between disease groups. These transcriptional programs were not reflected in conventional protein-level quantification or histopathological classification, supporting their interpretation as transcriptomic states rather than validated signaling activation. Importantly, they are not mutually exclusive and may co-exist within microglial populations.

snRNA-seq may underrepresent certain activation-associated transcripts, particularly cytoplasm-enriched genes [47]. Nevertheless, canonical robustly detected, supporting adequate capture of microglial transcriptional programs. Accordingly, conclusions are based on pathway-level patterns rather than single genes.

Differences in immune signaling may also influence therapeutic responses. In anti-DBN-positive patients, reduction or removal of anti-DBN antibodies may eliminate a disease relevant factor [11], potentially allowing partial functional recovery. In contrast, anti-GAD65-positive patients often show limited sustained benefit following AB reduction, suggesting more complex downstream mechanisms [48].

Cross-species comparisons with the OVA-CD8⁺ LE model reveal partially overlapping inflammatory transcriptional programs. However, this model represents an acute CD8 + T cell-driven system and does not recapitulate AB-associated human disease. Therefore, similarities should be interpreted as convergent inflammatory motifs rather than evidence of shared mechanisms or disease equivalence. In the mouse model, NF-κB-associated programs are prominent alongside interferon signaling, consistent with STAT1 activation downstream of IFN-γ pathways [49, 50]. In contrast, IFN-γ transcripts were largely absent in human snRNA-seq data, supporting temporal and spatial divergence between acute experimental and chronic human disease states.

**Fig. 6:**
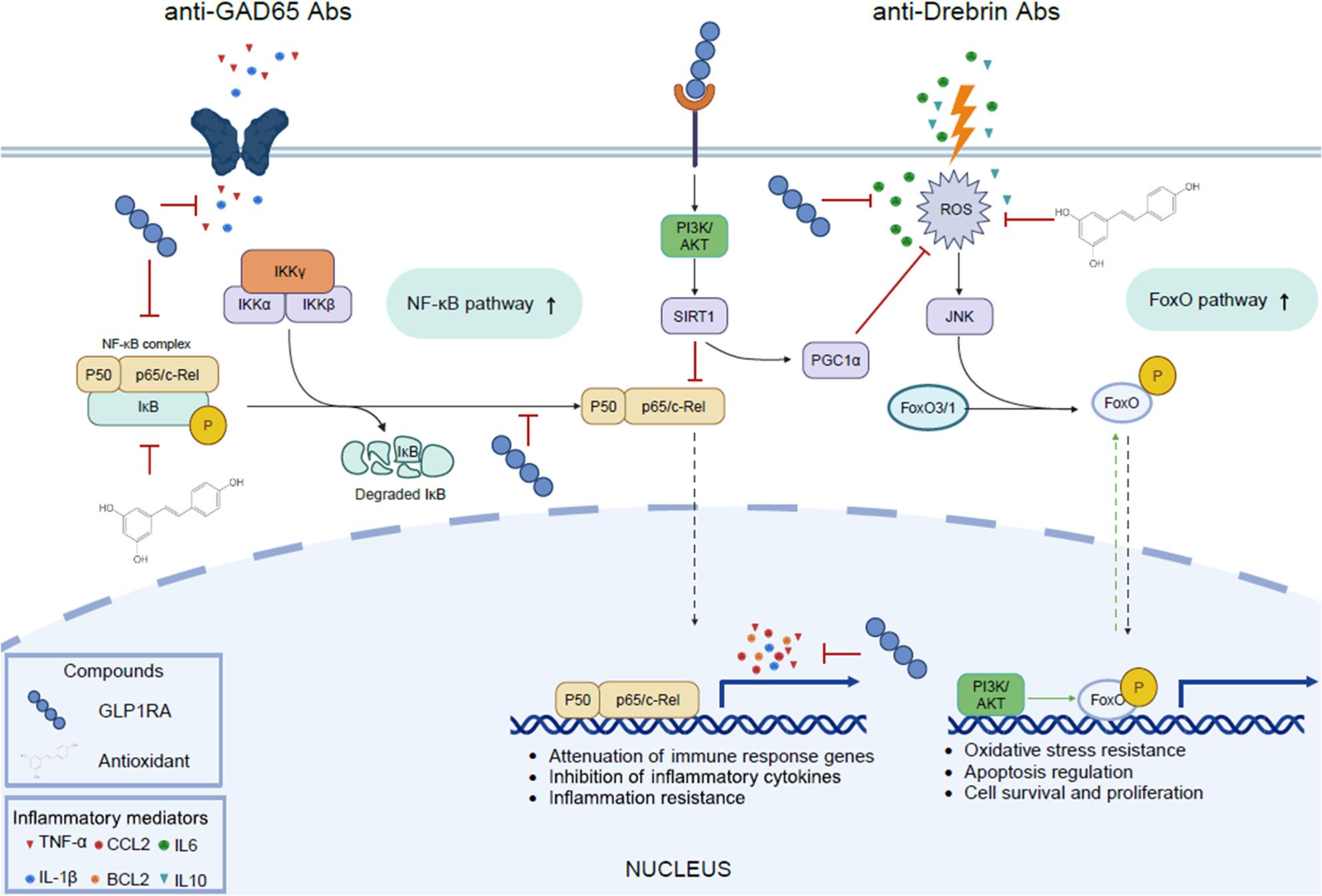
Conceptual model of anti-inflammatory mechanisms of resveratrol and GLP-1 receptor agonists (GLP-1RAs) in DBN- and GAD65-TLE. Natural antioxidants such as resveratrol inhibit nuclear factor-κB (NF-κB) signaling and reduce reactive oxygen species (ROS). Glucagon-like peptide-1 receptor agonists (GLP-1RAs) exert both direct and indirect anti-inflammatory effects by attenuating NF-κB signaling, including prevention of κBα (IκBα) degradation and suppression of pro-inflammatory mediators such as tumor necrosis factor-α (TNF-α), interleukin-6 (IL-6), interleukin-1β (IL-1β), and B-cell lymphoma-2 (BCL-2). In addition, GLP-1RAs activate the phosphoinositide 3-kinase/protein kinase B (PI3K/Akt) pathway, thereby modulating FOXO transcription factors under conditions of cellular stress, and promote activation NAD-dependent deacetylase sirtuin-1 (SIRT1). This signaling axis contributes to reduced ROS production via peroxisome proliferator-activated receptor gamma coactivator-1α (PGC-1α) and inhibition of NF-κB nuclear translocation. Together, these mechanisms highlight complementary anti-inflammatory pathways through which resveratrol and GLP-1RAs may modulate microglial signaling in DBN- and GAD65-TLE. Created with BioRender.com

Microglial NF-κB signaling is implicated in both neurodegenerative [51] and autoimmune diseases [33]. Persistent NF-κB activity may contribute to chronic inflammation and treatment resistance in anti-GAD65-positive TLE. Modulation of microglial inflammatory states has therefore been proposed as a potential therapeutic avenue, although this remains speculative [52]. Experimental compounds such as resveratrol and GLP-1 receptor agonists have been shown to influence NF-κB and FoxO-related inflammatory pathways and neuroprotective signaling in preclinical systems [53–55] (Fig. 6), but these findings remain indirect and not clinically validated.

Several limitations should be acknowledged. The small sample size (*n* = 2 per group), together with differences in age, sex distribution, disease duration, and treatment history, limits statistical power and precludes robust adjustment for confounding variables. In particular, the higher age at onset in the anti-DBN group raises the possibility that FoxO-associated transcriptional signatures may in part reflect age- or chronicity-related microglial states rather than disease-specific effects. Regression-based covariate adjustment was not feasible due to sample size constraints and cohort heterogeneity; therefore, SCTransform-based integration without explicit covariate regression was applied. As a results, residual confounding cannot be excluded. In addition, the limited number of T cell nuclei restricted CTL-specific downstream analyses.

Taken together, the data provide a spatially and transcriptionally resolved description of immune and glial cell states in rare autoimmune epilepsy tissue. However, given the limited cohort size and heterogeneity, the observed differences should be considered exploratory and hypothesis-generating rather than indicative of definitive disease-specific inflammatory programs. Nevertheless, seizure freedom following immunotherapy was observed numerically more frequently in the anti-DBN group (40% vs. 10%). This trend is consistent with the overall clinical impression that anti-DBN-associated TLE may exhibit greater immunotherapy responsiveness than anti-GAD65-associated disease and raises the possibility that the distinct transcriptional programs identified in the present study reflect biologically different inflammatory states. This hypothesis warrants investigation in larger cohorts.

### Material & methods

#### Standard protocol approvals, registrations, and patient consents

We confirm that we have read the Journal’s position on issues involved in ethical publication and affirm that this report is consistent with those guidelines. All procedures were conducted in accordance with the Declaration of Helsinki. Informed written consent was obtained from every patient according to the approvals of the local ethics committee (Nr. 229/18, 371/20, 504/20). All animal procedures were in accordance with the guidelines of the University Hospital Bonn, Animal-Care-Committee as well as the guidelines approved by the European Directive (2010/63/EU) on the protection of animals used for experimental purposes and ARRIVE guidelines.

#### Patients 

Serum and/or CSF GAD65 AB positivity was assessed using standardized clinical assays, and only patients with high-titre GAD65 autoimmunity were included. Given that all included cases fell within a uniformly high-titre range, quantitative differences in AB levels were not considered for further stratification or analysis. Similar high-titre inclusion criteria were applied for DBN-TLE patients; however, due to the lack of commercially available diagnostic assays, DBN AB detection was performed using a laboratory-developed assay. Cohort sizes are reported to provide context regarding the composition and representativeness of the study population; however, no statistical inference or interpretation of molecular findings was based on comparisons between cohort sizes.

To evaluate the treatment response, 22 patients with DBN- and 35 patients with GAD65-TLE were retrospectively analyzed for seizure outcome following anti-seizure medication (ASM), immunotherapy (IT), and/or epilepsy surgery. Pharmacoresistance to ASM was assessed according to the ILAE guidelines, which consider failure to achieve sustained seizure freedom (≥ 6 months of follow-up) with two appropriately selected and tolerated ASM regimens [25]. Response to IT was defined as seizure freedom, or a clinically meaningful improvement in seizure frequency at follow-up of at least nine months. Epilepsy surgery outcome was evaluated according to the ILAE-proposed Engel classification [56]. Additional clinical data of both cohorts are provided in Table 1.

#### Immunohistochemistry 

Surgically removed brain biopsy tissue was paraformaldehyde (PFA)-fixed, paraffin-embedded, sectioned (4 μm), and processed according to standard protocols [57] with antibodies described in supplementary Methods.

#### Single-nucleus isolation, library preparation, RNA sequencing, data analysis 

A total of eight hippocampal samples were used for snRNA sequencing. These included tissue from TLE patients who underwent amygdalo-hippocampectomy to remove the epileptic focus for seizure relief and were seropositive for anti-DBN (*n* = 2) or anti-GAD65 (*n* = 2). As controls, hippocampi of seronegative patients with HS (*n* = 2) and brain tissue of patients with lesion associated epilepsy (*n* = 2) were included. Following surgical resection, all brain tissue was immediately cryopreserved and stored at -80 °C until further processing. snRNAseq data were mapped to the human reference genome using Cell Ranger and imported into R for further QC and analyses (see Supplementary Methods for details). Samples were integrated using the SCT algorithm implemented in Seurat, accounting for mitochondrial reads and cell cycle effects, before clustering and performing differential expression nuclei-wise on the pooled nuclei for each phenotype (using the Wilcoxon Rank Sum test with Bonferroni correction). We evaluated age- and sex-regression approaches during data integration. However, given the limited sample size, these strategies resulted in unstable clustering and evidence of overcorrection. Therefore, SCTransform-based integration was used without explicit covariate regression. Detailed nuclei extraction, library preparation, RNA sequencing and data analysis (immune cell profiling, gene ontology, druggability) is described in supplementary Methods.

### Experimental animals, encephalitis induction, RNA isolation, library preparation, and bulk RNA-seq data analysis

All animal procedures were performed in accordance with European, national and institutional guidelines (for details see supplementary Methods). Encephalitis was induced in adult double transgenic (OT-I/RAG1^−/−^) mice using an AAV vector-based antigen transfer approach as previously described [26]. Mice hippocampi were dissected, and RNA isolated followed by library preparation and bulk RNA isolation and library preparation as described in supplementary material.

## Supplementary Information

Supplementary Material 1.

Supplementary Material 2.

Supplementary Material 3.

Supplementary Material 4.

Supplementary Material 5.

## Data Availability

The RNA-seq datasets generated and analyzed during this study are available in the Gene Expression Omnibus (GEO) repository under the accession numbers **GSE303810** (Human snRNA-seq dataset) and **GSE303749** (Mouse RNA-seq dataset). To facilitate peer review while the records remain private, the following secure tokens have been generated: - For **GSE303810** : mzsragswzvexlqv - For **GSE303749** : anobckyqptwvdmz
