# Supplementary material for: Forkhead box versus NF-κB hippocampal snRNA-seq profiles distinguish anti-Drebrin- and anti-GAD65-positive encephalitis
Source: J Neuroinflammation. 2026 Jul 7;23:232. doi: 10.1186/s12974-026-03951-8 (PMC13343577; doi:10.1186/s12974-026-03951-8)
Supplement: Supplementary file 1 — Supplementary Material 1. [file 12974_2026_3951_MOESM1_ESM.docx]

**Supplementary figures**

**Forkhead box versus NF-κB hippocampal snRNA-seq profiles distinguish anti-Drebrin- and anti-GAD65-positive encephalitis**

**
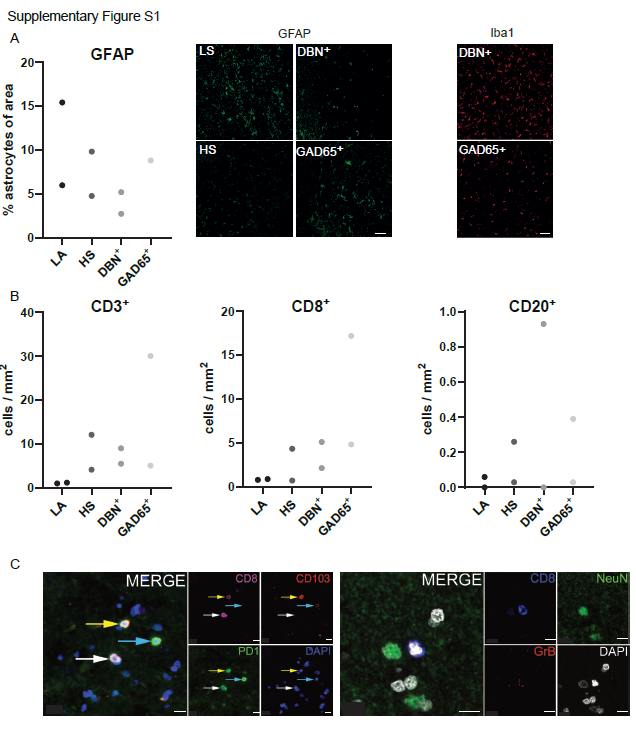
**

**Supplementary Figure S1: Immune cell repertoire in sequenced DBN-TLE and GAD65-TLE cases.** Immune cell composition was assessed by immunohistochemistry to determine the presence of (A) astrocytes (GFAP) and microglia (Iba1), (B) CD3⁺ and CD8⁺ T cells, as well as CD20⁺ B cells across patient groups. (C) Expression of exhaustion- and activation-associated markers, including CD103, PD-1, and granzyme B in CD8^+^ T cells.


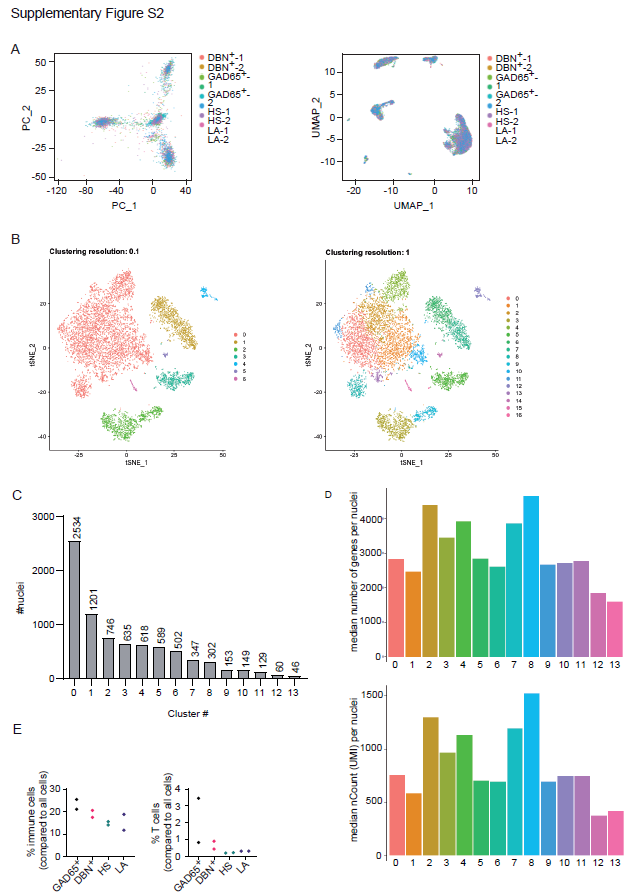


**Supplementary Figure S2:** **Uniform nuclei integration among all sample groups.** Nuclei integration of anti-Drebrin-positive (n = 2), anti-GAD65-positive TLE patients (n = 2), lesion-associated epilepsy (LA; n = 2) and patients with hippocampal sclerosis (HS; n = 2) using Principal Component Analysis (PCA, left panel) and Uniform Manifold Approximation and Projection (UMAP, right panel) plot of all recovered nuclei from the eight individual patients. (**B**) tSNE plot at low resolution (0.1) showing 7 individual clusters with distinct transcriptional profiles, and clustering at high resolution (1.0) showing 17 individual clusters. (**C**) The absolute numbers of sequenced nuclei within each of the 14 clusters. (**D**) Median number of genes per nuclei (upper panel) and median nCount (UMI) per nuclei within the 14 clusters. (**E**) Percentage of immune cells (left panel) and T cells (right panel) compared to the total number of cells in the four clinical subgroups.


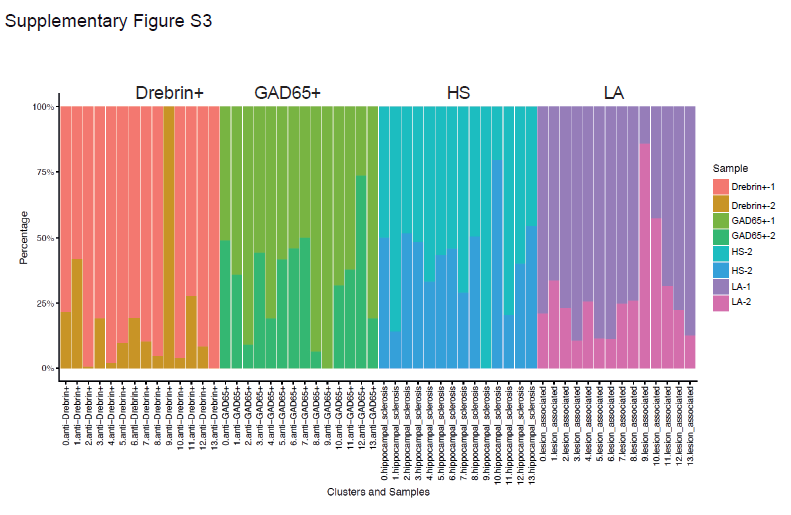


**Supplementary Figure S3.** Per-patient contribution to snRNA-seq clusters.
Bar plots show the percentage contribution of nuclei from each individual patient to each of the 13 identified clusters. Patients are grouped by clinical subgroup: anti-Drebrin-positive (1, 2), anti-GAD65-positive (7, 8), hippocampal sclerosis controls (5, 6), and lesion-associated controls (3, 4). Each bar represents a single cluster, and the stacked segments indicate the proportion of nuclei derived from each patient.


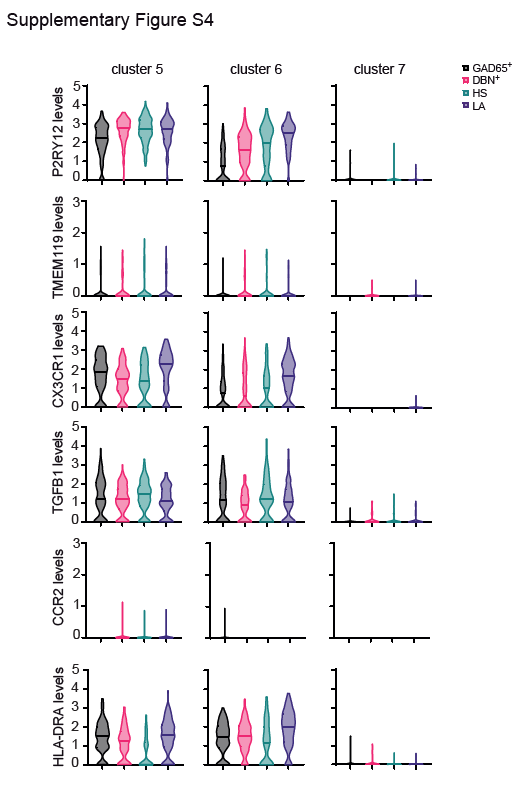


**Supplementary Figure S4:** **Microglia and infiltrating myeloid transcript signatures in hippocampal biopsies of anti-GAD65 and anti-Drebrin-positive TLE patients versus controls.** Expression levels of P2RY12, TMEM119, CX3CR1, TGFB1, CCR2 and HLA-DRA in immune clusters 5, 6 and 7 in the four clinical subgroups. GAD65+ = anti-GAD65-positive TLE, DBN+ = anti-Drebrin-positive TLE, HS = hippocampus sclerosis, LA = lesion-associated patients.


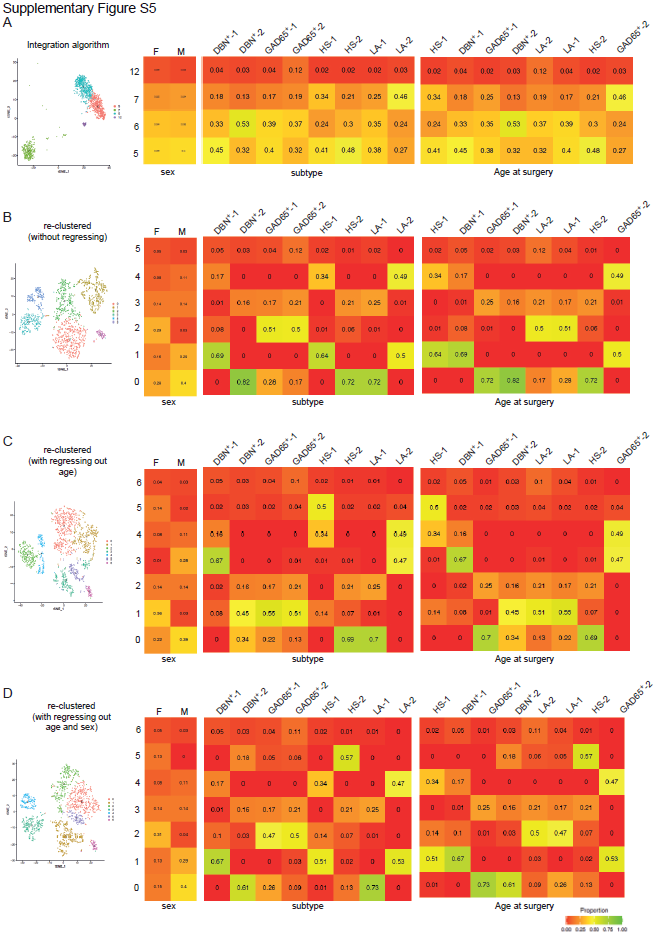


**Supplementary Figure S5:** **Re-clustering of the immune-populations does not improve confounding effects of age or sex.** Clustering of the immune clusters 5, 6, 7 and 12, on sex, clinical subtype and age at surgery of our dataset obtained using (**A**) the integration algorithm, (**B**) re-clustering without regressing, (**C**) re-clustering with regressing out age, and (**D**) re-clustering with regressing out age and sex. GAD65+ = anti-GAD65-positive TLE, DBN+ = anti-Drebrin-positive TLE, HS = hippocampus sclerosis, LA = lesion-associated patients. F = Female, M = Male.


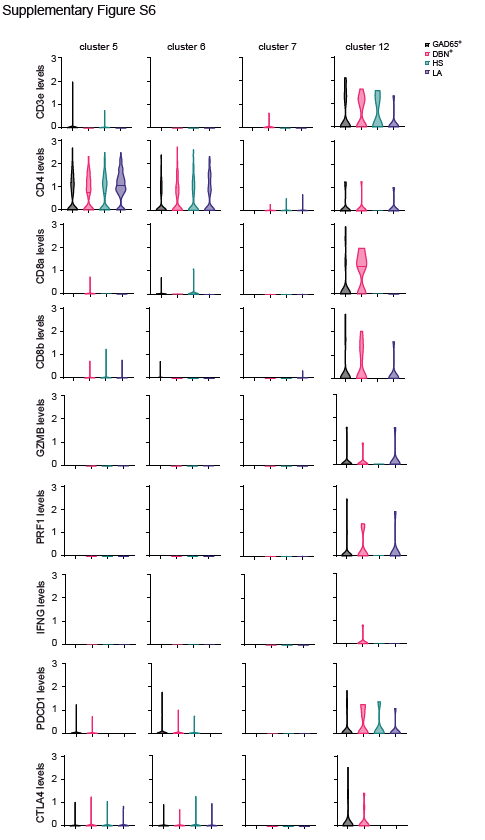


**Supplementary Figure S6:** **Immune cell transcript signatures in hippocampal biopsies of anti-GAD65 and anti-Drebrin-positive TLE patients versus controls.** Expression levels of the immune markers CD3e, CD4, CD8a, CD8b, GZMB, PRF1, IFNG, PDCD1 and CTLA4 in clusters 5, 6, 7 and 12 in the four clinical subgroups. GAD65+ = anti-GAD65-positive TLE, DBN+ = anti-Drebrin-positive TLE, HS = hippocampus sclerosis, LA = lesion-associated patients.


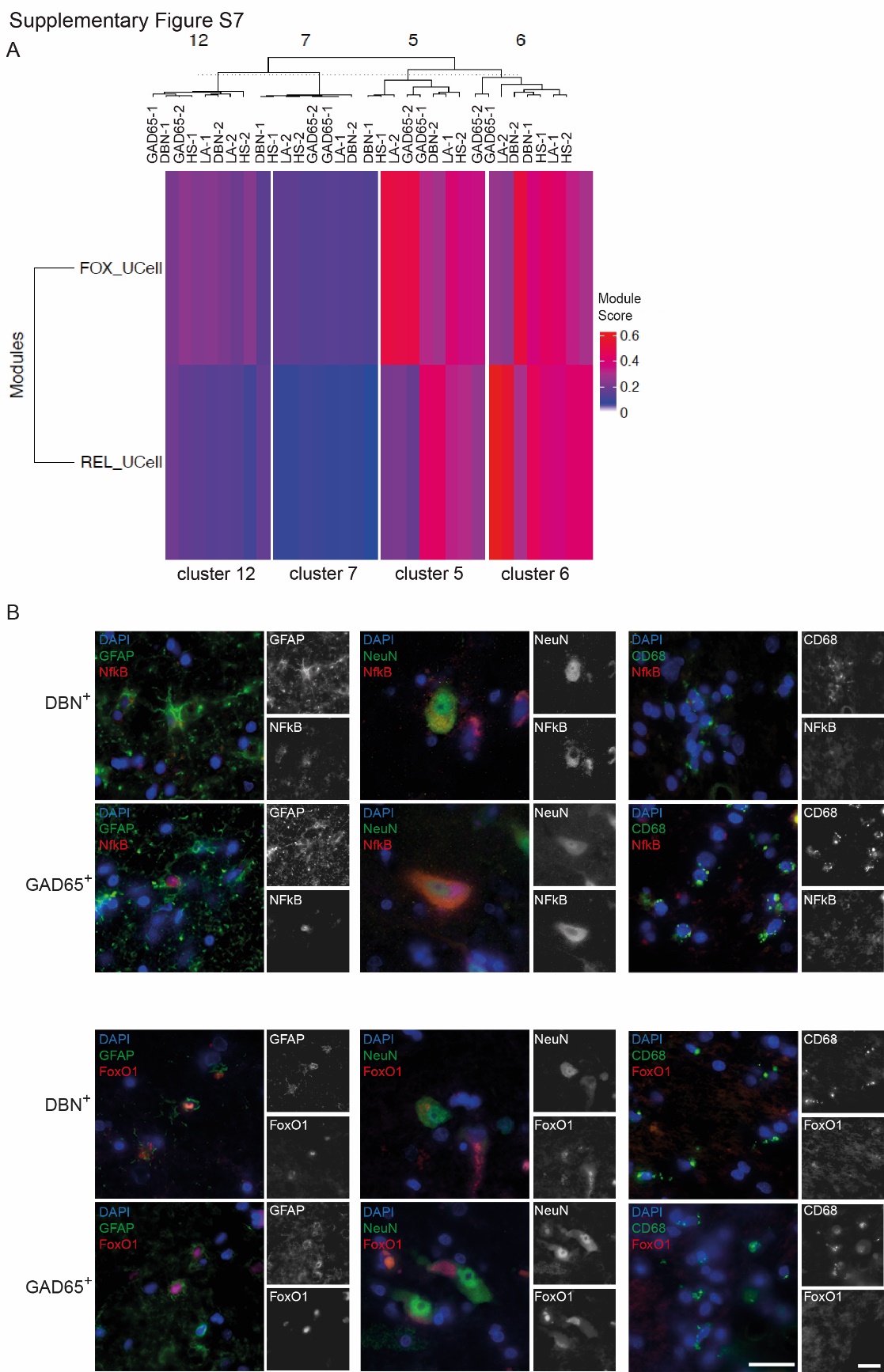


**Supplementary Figure S7. Module scores of FoxO- and NF-κB–associated gene sets across clusters and samples. (A)** Heatmap showing per-sample module scores for FoxO-associated (FOX_UCell) and NF-κB–associated (REL_UCell) gene signatures across selected clusters (clusters 5, 6, 7 and 12). Samples include anti-GAD65 (GAD65-1/2), anti-Drebrin (DBN-1/2), hippocampal sclerosis controls (HS-1/2), and lesion-associated controls (LA-1/2). Columns represent individual samples grouped by cluster, with hierarchical clustering indicated above the heatmap. Rows correspond to pathway-specific module scores calculated using UCell. Color scale indicates relative enrichment (blue, low; red, high). **(B**) Representative NF-κB and FoxO labelling from surgically removed brain tissue of DBN- and GAD65-TLE patients showing ubiquitous expression in neurons (NeuN), astrocytic (GFAP), and microglial cells (CD68).


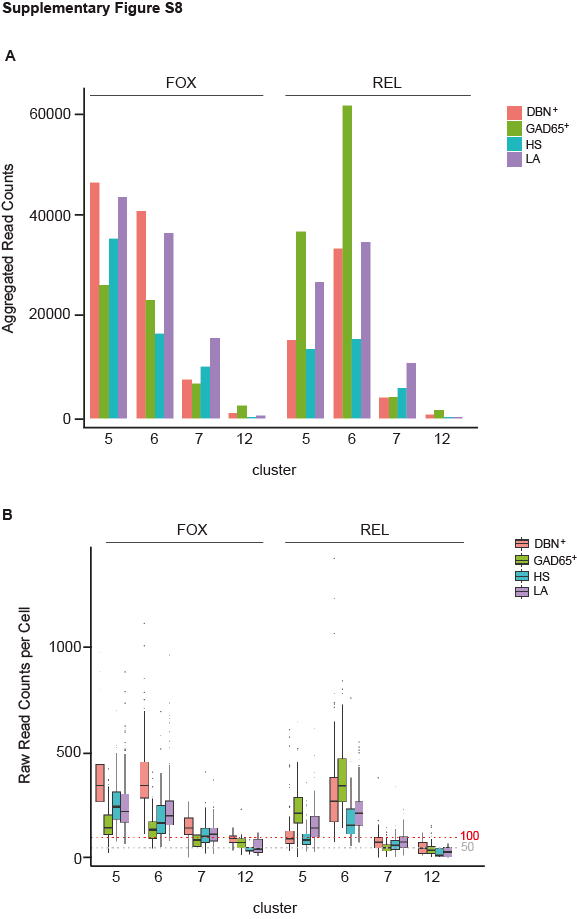


**Supplementary Figure S8. Detection of REL/RELB- and FOXO1/FOXN2-associated transcripts across immune clusters.** (**A**) Aggregated read counts of genes included in the REL/RELB and FOXO1/FOXN2 target gene analyses across immune cell clusters identified by snRNA-seq. Total read counts were calculated by summing reads across all genes belonging to the respective transcription factor-associated gene sets within each cluster. Clusters 5 and 6, representing the largest microglial-enriched populations, showed the highest transcript abundance, whereas lower counts were observed in smaller immune clusters. (**B**) Distribution of raw transcript counts for genes included in the REL/RELB and FOXO1/FOXN2 target gene analyses at the single-cell level across samples and immune clusters.


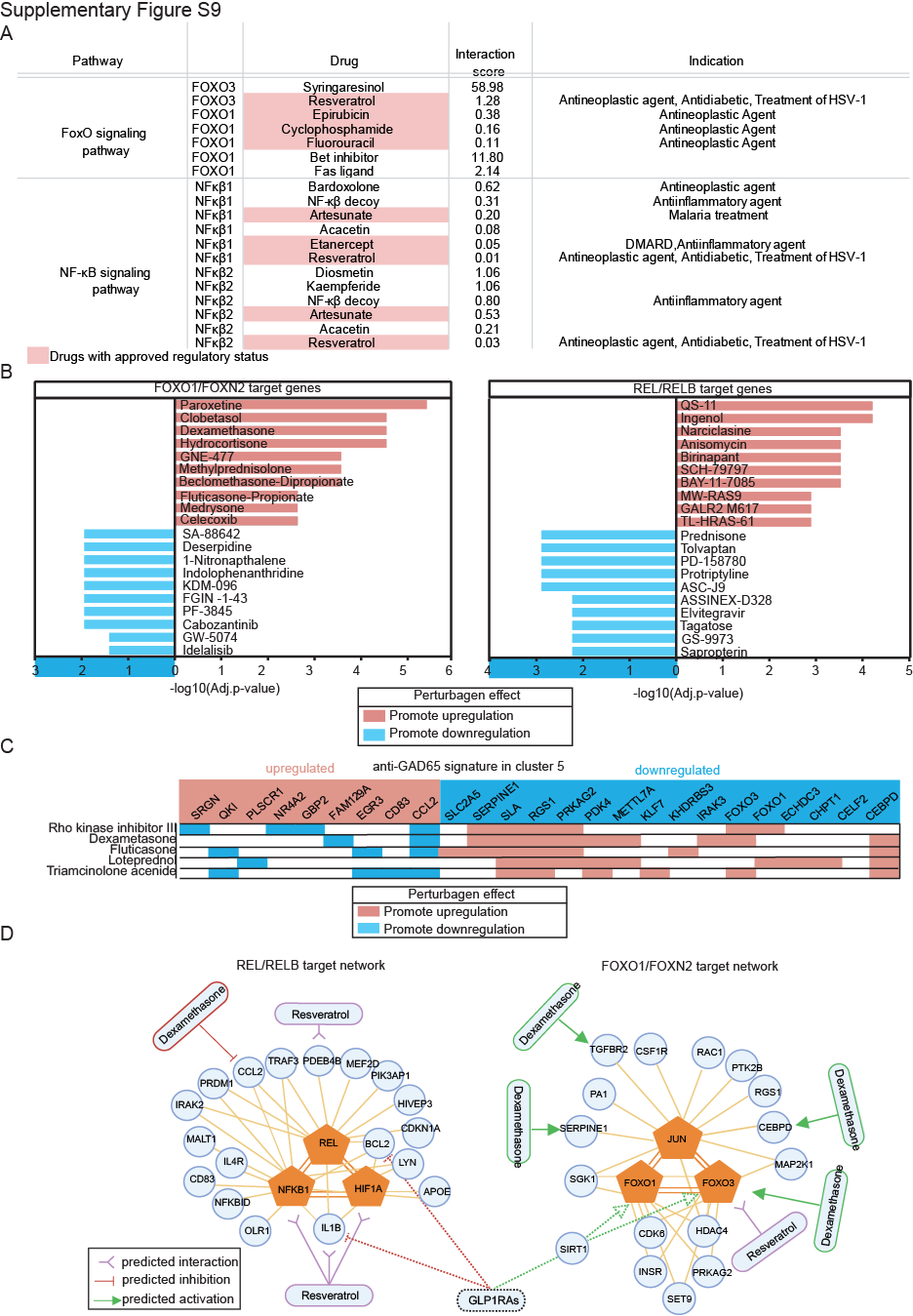


**Supplementary Figure S9. Drug-gene interactions linked to the main altered signaling pathways.** **(A)** Key transcription factors associated with the FoxO and NF-κB pathways for drugs with the highest interaction scores. **(B)** Target genes for FOXO1/FOXN2 and REL/RELB found in the LINCS L1000 Chemical Perturbation Consensus Signature database (https://maayanlab.cloud/Enrichr/), indicating predicted increase or decrease of genes associated with the most relevant chemical compounds. **(C)** Small molecules predicted (https://maayanlab.cloud/L1000CDS2/) to reverse the gene signature of the anti-GAD65 profile (cluster 5) and the upregulation/downregulation of target genes. **(D)** Graphical representation of the STRING (<https://string-db.org/>) interactions between the REL/RELB TFs and anti-Drebrin-specific DEGs (left panel), and the interactions between the FOXO1/FOXN2 TFs and the anti-GAD65-specific DEGs in the immune clusters (right panel). Only genes directly linked to the main TFs are depicted. Gene-drug interactions are observed with the highest ranked drugs. GAD65^+^ = anti-GAD65-positive TLE, DBN+ = anti-Drebrin-positive TLE, HS = hippocampus sclerosis, LA = lesion-associated patients.


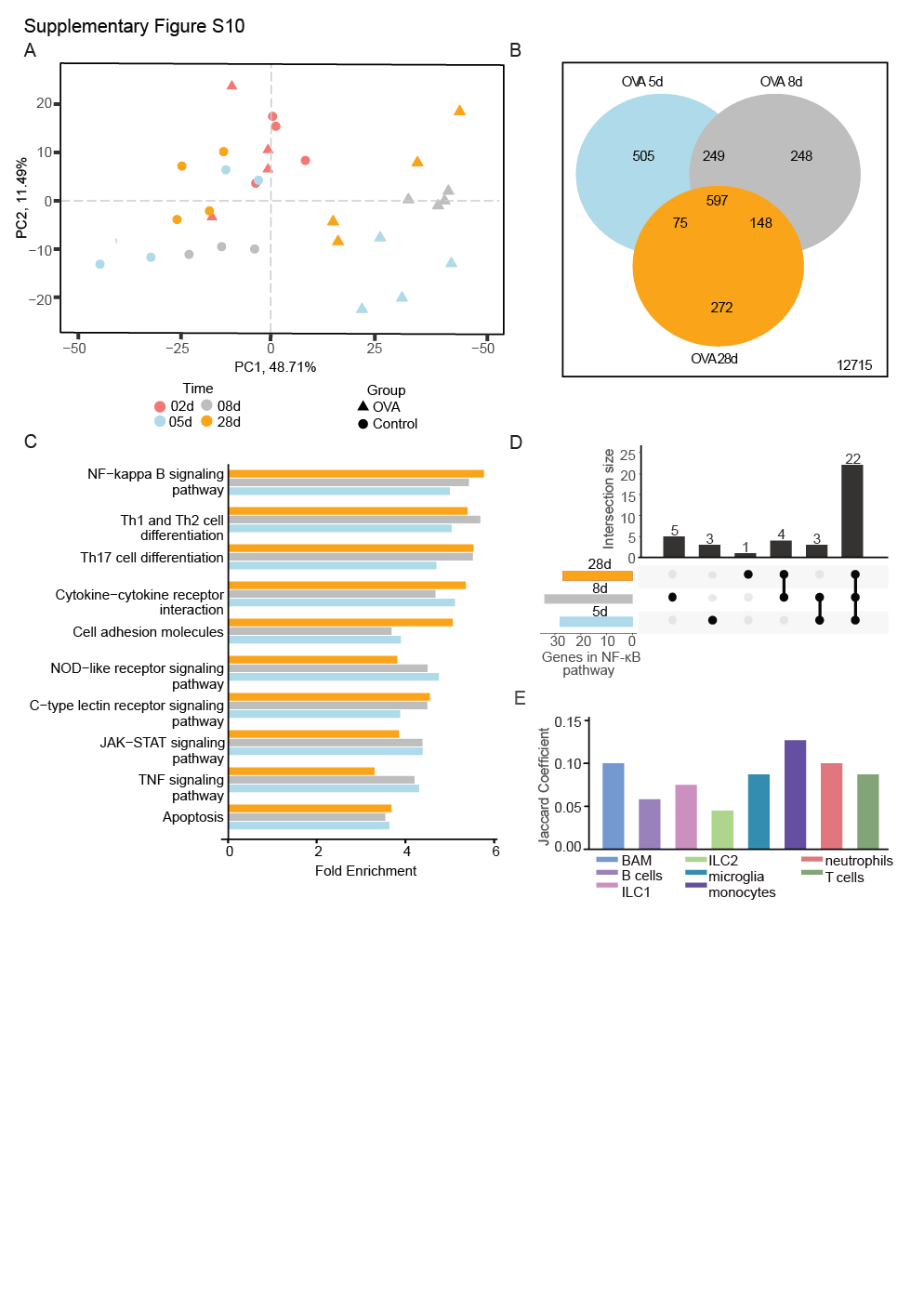


**Supplementary Figure S10. Transcriptomic alterations during T cell–driven encephalitis correlate with increased NF-κB signaling. (A)** Principal component analysis (PCA) reveals two main clusters corresponding to T cell–induced encephalitis and control samples. Pronounced transcriptional variation is observed between groups. **(B)** Venn diagram showing a high overlap of differentially expressed genes (DEGs; FDR ≤ 0.05; |Log₂FC| > 0.66) across T cell–driven encephalitis at 5, 8, and 28 days after vector-based antigen transfer, compared with matched controls. **(C) T**op 5 enriched (FDR<0.01) KEGG pathways among upregulated DEGs (FDR ≤ 0.05; Log₂FC| > 0.66) across time points, highlighting sustained inflammatory and immune-related signaling. **(D)** UpSet plots illustrating substantial overlap of upregulated DEGs mapping to the NF-κB signaling pathway across disease stages. The canonical murine NF-κB pathway (mmu04064) comprises 97 genes, as retrieved using pathfindR. **(E) ***Jaccard coefficient analysis of genes upregulated during early T cell–induced encephalitis (5 days), derived from single-cell CITEseq data reported by *Golomb et al*. ^21^.

**Supplementary Table 1:** **Patient- and nucleus-specific expression levels of immune genes in clusters 5, 6, 7 and 12.**

**Supplementary Table 2:** **Differentially Expressed Genes (DEGs) between anti-GAD65- and anti-Drebrin-positive patients in main the immune Clusters.** DEGs are defined based on a False Discovery Rate (FDR) ≤ 0.1 for the comparisons within each cluster (clusters 5, 6, and 7) between the anti-Drebrin and anti-GAD65 conditions. The average log2 fold change (Log2FC) indicates the identity of the DEG, with Log2FC ≤ 0 corresponding to anti-Drebrin and Log2FC ≥ 0 corresponding to anti-GAD.

**Supplementary Table 3: Most prominent gene expression changes at 2, 5, 8, and 28 days after vector-based antigen transfer and initiation of limbic encephalitis.** The Differentially Expressed genes (DEGs) between rAAV-OVA and rAAV-control samples are defined by FDR ≤ 0.05. The direction is defined by Log2FC; for the increased genes Log2FC ≥ 0.66 (red) and for the decreased ones Log2FC ≤ -0.66 (blue).
