# Supplementary material for: Forkhead box versus NF-κB hippocampal snRNA-seq profiles distinguish anti-Drebrin- and anti-GAD65-positive encephalitis
Source: J Neuroinflammation. 2026 Jul 7;23:232. doi: 10.1186/s12974-026-03951-8 (PMC13343577; doi:10.1186/s12974-026-03951-8)
Supplement: Supplementary file 2 — Supplementary Material 2. [file 12974_2026_3951_MOESM2_ESM.docx]

**Supplementary Material**

**Forkhead box versus NF-κB hippocampal snRNA-seq profiles distinguish anti-Drebrin- and anti-GAD65-positive encephalitis**

**Running head:**

Transcript signatures in Drebrin and GAD65 encephalitis

Karen M.J. van Loo^1,*^, Daniel S. Galvis-Montes^2,*^, Annika Breuer^2^, Juliane L. Berns^2^, Chiara Hummel^2^, Tobias Baumgartner^2^, Moritz Freyberg^2^, Katharina M. Mair^3,^ Jan Bauer^3^, Theodor Rüber^2,4^, Ashley J. van Waardenberg^5^, Motaz Hamed^6^, Valeri Borger^6^, Hartmut Vatter^6^, Rainer Surges^2^, Susanne Schoch^2,7^, Albert J. Becker^7#^, Julika Pitsch^2#^

# ^1^Department of Epileptology, Neurology, RWTH Aachen University, Aachen, Germany,

# ^2^Department of Epileptology, University Hospital Bonn, Bonn, Germany,

# ^3^Department of Neuroimmunology, Centre for Brain Research, Medical University of Vienna, Vienna, Austria,

# ^4^Department of Neuroradiology, University Hospital Bonn, Bonn, Germany,

# ^5^i-Synapse, Cairns, Australia,

# ^6^Clinic for Neurosurgery, University Hospital Bonn, Bonn, Germany,

# ^7^Institute of Cellular Neurosciences II, University Hospital Bonn, Bonn, Germany

**Immunohistochemistry**

Antibodies specific for neuronal nuclei (NeuN) (Millipore, MAB377, 1:1000), glial fibrillary acidic protein (GFAP) (Dako Denmark A/S, Z0334, 1:100), CD68 (Dako Denmark A/S, M0814, 1:50), CD3 (Leica Biosystems, NCL-L-CD3-565, 1:50), and CD8 (Dako Denmark A/S, M7103, 1:50), were used over night at 4°C. After washing, secondary biotinylated antibody (Vectorlabs, anti-human BA3000 and anti-mouse BA9200) was applied at room temperature (RT) for 2 h (1:200). Incubation with avidin-biotin-peroxidase (Vectorlabs, PK6100) followed for 30 min at RT. Antibody binding was visualized using 3,3’-diaminobenzidine (DAB; Sigma-Aldrich, D4293-50SET). Before slides were covered, cell nuclei were counterstained with haematoxylin. Afterwards, images were captured with Keyence BZ-X800.

**Single nuclei isolation, library preparation, RNA sequencing**

Nuclei were extracted in lysis buffer (nuclei extraction buffer (Miltenyi Biotex) with RNase inhibitor (Promega, N2615)) using a gentleMACS dissociator (Miltenyi Biotec). The nuclei suspension was then filtered through a MACS SmartStrainer (70 µm) with the strainer rinsed with lysis buffer. The filtered suspension was centrifuged at 300 x g for 5 min at 4 °C. The pellet was resuspended in 3 ml resuspension buffer (PBS + 0.04 % BSA + 0.2U/ul RNAse inhibitor), and filtered through a MACS SmartStrainer (30 µm). The collected nuclei suspension was immediately processed for debris removal using density gradient centrifugation. For debris removal, the nuclei suspension was centrifuged at 300 x g for 10 min at 4 °C, resuspended in 900 µl PBS, and gently layered with 355 µl debris removal solution (Milteny), followed by 4 ml PBS. Density gradient centrifugation was performed at 3000 x g for 10 min at 4 °C. The two top phases were aspirated, and the tube was filled up with 15 ml PBS, inverted to mix, and centrifuged again at 1000 x g for 10 min at 4°C. The final nuclei pellet was resuspended in 500 µl resuspension buffer and stained with DAPI (1.7µg/ml, Sigma-Aldrich). Nuclei were sorted using BD FACSAria III (100 µm nozzle size, 20psi, 4°C). Nuclei were prepared using the dual-index Chromium Next GEM Single Cell 3’ Kit v3.1 (10xGenomics) with a target nuclei load of approximately 16,000 per sample. Sequencing was performed on a Novaseq6000 (28/8/91, Illumina) using S2 flow cell with a target of 50,000 reads per nucleus following standard protocols.

**Analysis of snRNA-seq data**

Mapping of snRNA-data was performed using Cell Ranger version 6.1.2 ^1^. The “GRCh38-3.0.0” reference transcriptome for the human genome was downloaded from 10xgenomics here: https://cf.10xgenomics.com/supp/cell-exp/refdata-cellranger-GRCh38-3.0.0.tar.gz. Fastq files of the raw sequencing data for each sample were input into the cellranger “count” subcommand, with parameters –expect set to 7000 and run two times, firstly with “—include-introns” and secondly without “—include-introns”.

Raw feature count matrices generated by Cell Ranger were imported into R (version 4.0.5) using the ‘read10xCounts’ function (Seurat version 4.0.2) ^2^, followed by conversion to a SeuratObject using the ‘CreateSeuratObject’ function. DIEM (version 2.3.0) was used to infer which nuclei were contaminated, based on mitochondrial read counts, using a threshold score of “0.5” (call_targets function) ^3^. Cell cycle state was scored using the Seurat function ‘CellCycleScoring’, (using Seurat provided 2019 updated genes for s and g2m features) ^4^ after initial normalization, ‘NormalizeData’, using default parameters. For filtering of cells based on mitochondrial and ribosomal read counts, the ‘PercentageFeatureSet’ function of Sureat was used to determine the percentage of reads that mapped to ‘MT-*’ and ‘RPS* or RPL*’ genes respectively. Furthermore, counts from intron and exon mapped 10X transcriptomes were computed as a ratio for each nucleus, to determine nuclei with poor pre-RNA transcript enrichment. After inspection of QC plots, nuclei were then filtered if the number of genes detected ‘nFeature_RNA’ was >= 500 and <= 8000, percentage of reads mapped to mitochochondria genes <= 5, percentage of reads mapped to ribosomal genes <= 5 and intron versus exon ratio was >= 1.5. Doublet identification was conducted on each dataset to remove multiplets using ‘DoubletFinder” version 2.0.3 ^5^, downloaded from: “github - chris-mcginnis-ucsf/DoubletFinder”. The number of expected doublets was set to 4% on expected counts of 8,000 loaded cells and parameters pN=0.25, pK=0.09, using 30 principal components. Data were transformed using ‘SCTransform’ ^6^, with the effect of cell cycle and mitochondrial expression regressed out prior to integration of all nuclei into a single tSNE projection by sequential calling of ‘SelectIntegrationFeatures’, ‘PrepSCTIntegration’, ‘FindIntegrationAnchors’, and ‘IntegrateData’. ‘anchor.features’ were set to all features and ‘normalization.method’ to SCT.

All features (transcripts) were then used for resolving clusters. Principal Component Analysis (PCA) dimensionality reduction (RunPCA) on the first 30 principal components, followed by tSNE generation (RuntSNE) using the prior PCA reductions. Nearest-neighbor graphs were then constructed using the FindNeighbors on the PCA reductions and clusters determined using FindClusters resolution of 0.6, identifying 14 clusters. For assigning markers to each cluster, the ‘FindMarkers’ function was called with each cluster selected as ‘ident.1’, using the remaining clusters as background, using the default Wilcoxon Rank Sum test with Bonferroni correction applied for multiple hypothesis testing. ‘FindMarkers’ was also called for each condition (being the combined nuclei for each phenotype) within each cluster to test for differential expression between conditions, here ‘ident.1’ and ‘ident.2’ were assigned to nuclei belonging to a condition within each cluster. As the samples were of mixed sex, ‘FindMarkers’ was called on all nuclei that belonged to male or female to identify genes that were differentially expressed between sex irrespective of the condition. Genes were considered sex associated if there was significant (p-value <= 0.1) differential expression between sexes and removed in subsequent analysis. Differential expression between the groups was visualized using the DeepVenn deep learning framework Tensorflow.js ^7^.

For annotation of clusters according to immune cell type, Cite-seq data of corresponding to immune cell data from whole brain from Golomb et al. was downloaded ^8^. Firstly, of the 11 clusters identified in Golomb, the proportion of cells annotated to each of the immune cell types were assessed for specificity. Non-specific clusters (cluster 1, 10, 11, 3), that is where there was a lack of cell type enrichment or mixed annotations, were ignored for subsequent analysis. Input was the top 1000 transcripts identified as cell cluster markers from the snRNA-seq differential expression analysis performed and described above for each cluster. The jaccard coefficient was then calculated for each of the clusters, being ratio of the intersect of cell markers for each cluster identified in Golomb et al, with the top 1000 markers from each snRNA-seq cluster versus the union of markers from both studies. This identified clusters 5, 6, 7 and 12 as demonstrating enrichment for immune cell markers. Nuclei belonging to each of these clusters were isolated and reanalysed to identifying sub-clusters, following the same procedure for the overall analysis, identifying 6 clusters (excluding cluster 12 which had too few nuclei for further subclustering). For each of these sub-clusters, Golomb’s cite-seq data was further used to determine cell type enrichment for each cluster. For each of the sub-clusters, differential expression between phenotypes, as described above, was further performed.

**Drug-gene interaction analysis and druggability analysis**

The potential druggability of genes of interest was assessed using the Drug-Gene Interaction Database (DGIdb) ^9^. This tool allows genes of interest to be correlated with respect to known drug-gene interactions and potential druggability. The DGIdb calculates a scoring metric for ranking based on the interaction score. Interaction score, literature reports and pharmacological indications were used as selection criteria. Additionally, the L1000CDS² tool was employed to identify potential molecular targets and drug candidates predicted to reverse or mimic the input signature. Enrichr was used to determine the specific gene profiles associated with perturbagens ^10^. The dataset underlying the L1000CDS² tool consists of LINCS L1000 small molecule expression profiles, generated by the Connectivity Map Group at the Broad Institute. The L1000 Connectivity Map is part of the Library of Integrated Network-based Cellular Signatures (LINCS) project, which profiles gene expression changes following pharmacologic or genetic perturbations in cell lines ^11^.

**Experimental animals and encephalitis induction**

All animal procedures were designed and performed to minimise pain and suffering and to reduce the number of animals used in accordance with European, national and institutional guidelines (European Parliament and Council guidelines on the protection of animals used for scientific purposes, European Directive (2010/63/EU) and ARRIVE guidelines). The study protocol was approved by the Landesamt für Natur, Umwelt und Verbraucherschutz (LANUV) of North Rhine-Westphalia, Germany. All mice were housed in a humidity (55 ± 10%) and temperature (22 ± 2°C) controlled environment under a 12-hour light-dark cycle (light cycle 7 am to 7 pm) with water and food ad libitum and nest material (Nestlets, Ancare, USA). Mice were allowed to acclimate to the facility for at least seven days prior to each treatment.

**For encephalitis induction,** adult transgenic OT-I/RAG1^-/-^ mice were used. The transgenic OT-I mouse strain is homozygous for a transgene that encodes a T cell receptor specific for chicken OVA 257–264, presented by the MHCI molecule H-2Kb. Additionally, these mice are deficient in the recombination activating gene 1 (RAG1) gene and therefore do not develop any mature T or B cells expressing endogenous receptors. Adult OT-I/RAG1^-/-^ mice were bilaterally injection with rAAV-OVA (rAAV-hSyn-OVA-mCherry; rAAV-OVA group) or a control vector carrying a fluorescent marker (rAAV-hSyn-mCherry; mCherry (control) group) into both dorsal CA1 hippocampal regions (stereotactic coordinates relative to bregma14: −2 mm anteroposterior [AP], −1.5 mm mediolateral [ML], −1.5 mm dorsoventral [DV]; 1 μl of viral suspension). All injected mice received analgesic treatment (5 mg/kg ketoprofen, subcutaneous; Rifen, Vetoquinol) before surgery and once daily for 3 consecutive days.

**RNA isolation and library preparation**

Mice were decapitated under deep isoflurane anesthesia (Forene, Abbott GmbH, Germany), after 2, 5, 8 and 28 days of rAAV-OVA or rAAV-mCherry injection. Brains were quickly removed and the left hippocampus was dissected. Total RNA was isolated and purified using the RNeasy micro kit (Qiagen) according to the manufacturer’s protocol. The quality and integrity of total RNA was assessed using a Tapestation 2200 (Agilent Technologies). RNA was considered as intact with an RNA Integrity Number (RINe) > 7. High purity RNA was considered with a OD260/280=1.8-2.1 and OD260/230 > 1.5 values. After purification, mRNA was used for library preparation using the TruSeq RNA Library Prep (Illumina) according to the manufacturer’s protocol. Sequencing was performed on a NovaSeq 6000 System (Illumina) using cycle paired-end sequencing.

**Bulk RNA-seq data analysis**

Fastq files were pre-processed using fastp 0.23.2 ^12^ to eliminate low quality reads and trim adapters ($ fastp -i ../path/to/in_R1.fastq.gz -I ../path/to/in_R2.fastq.gz -o ../path/to/out_R1.fastq.gz -O ../path/to/out_R2.fastq.gz --dont_overwrite --dup_calc_accuracy 6). Transcriptome and genome fasta files from GENCODE M29 ^13^ were used to build an index file with Salmon 1.8.0 ^14^ with the genome as a decoy to reduce spurious mapping ($ salmon index -t path/to/gentrome_m29.fa.gz -d path/to/decoys.txt -i path/to/salmon_index_m29 –gencode). Salmon ^14^ was then used for quantification of the pre-processed reads with this index ($ salmon -i path/to/salmon_index_m29 -l A -1 path/to/out_R1.fastq.gz -2 path/to/out_R2.fastq.gz --validateMappings --gcBias -o path/to/quant). Quantification data was imported via tximeta 1.14.1 ^15^ and transcripts summarized to the gene level. After filtering for genes with ≥ 15 counts in ≥ 15 samples (out of a total of 31 samples), the DESeq2 1.36.0 **^15^** function DESeq was run for differential expression testing. Additional batch correction was accounted for by two surrogate variables determined by sva 3.44.0 **^16^**. The ashr 2.2-54 **^17^** method was used for obtaining FDR-adjusted p-values (Benjamini and Hochberg 1995) and log2 fold-changes (LFCs) with shrinkage (to avoid overestimation for genes with higher uncertainty, such as low count genes). Genes were considered differentially expressed at FDR ≤ 0.05 and an absolute |Log2FC| ≥ 0.66. For downstream analyses other than differential expression testing, the variance-stabilizing transformation vst function by DESeq2 **^15^,** which for genes with high counts asymptotically equals a log2-transformation, was applied to the matrix of normalized gene counts. This resulting matrix underwent the limma 3.52.4 **^18^** function removeBatchEffect with the same statistical model used for differential expression testing.

**Gene ontology and kyoto encyclopedia of genes and genomes (KEGG) enrichment analysis**

Kyoto Encyclopedia of genes and genomes (KEGG) ^19^ pathway enrichment was performed using ShinyGO 0.80 ^20, 21^. KEGG pathway enrichment analysis was conducted to identify pathways associated with upregulated differentially expressed genes (DEGs) at 5, 8, and 28 days in the T cell–induced LE mouse model . Pathways were considered significant at FDR<0.05,;redundant pathways or those not associated with DEGs were excluded from the analysis. To interpret the molecular function and biological process of the DEGs, Gene Ontology (GO) enrichment analysis was performed using the GO enrichment analysis and visualization tool (GOrilla) ^22, 23^ and IDEP 2.0 ^20, 24^ with process ontology settings. Enrichment p-values were corrected for multiple testing using the Benjamini–Hochberg FDR correction, and pathways with an adjusted FDR ≤ 0.05 were considered significant. Transcription factor (TF) binding enrichment was performed using ChEA3, prioritizing TFs based on the overlap between DEG and TF targets assembled from ENCODE, ReMap, TTRUST 2019 (Transcription Regulatory Relationships Unraveled by Sentense-based Text mining) and TTRUST individual publications; co-expression of TFs with other genes based on processed RNA-seq from TRANSFAC and JASPAR databases ^10, 25, 26, 27^. For ChEA3, cluster-specific expressed genes were used as input, with the expressed-gene background for statistical correction. TFs with target sets <10 or >500 genes were excluded, and FDR-corrected p-values of 0.05 were considered significant. GSEA plot generation was performed by using the Bioconductor package org.Mm.eg.db (version 3.21.0) and the bitr() function from clusterProfiler (version 4.17.0) (Wu et al., 2021) (PMID: 34557778). DEGs from the T cell–induced LE mouse model (5d) were ranked by descending log₂FC values for gene set enrichment analysis. NF-κB-related gene sets were retrieved from the Biocarta collection in the msigdbr package (version 24.1.0). Gene Set Enrichment Analysis (GSEA) was performed using the GSEA() function from clusterProfiler with a significance cutoff of p-value < 0.1. Visualization of the top enriched NF-κB pathway was generated using the gseaplot2() function from enrichplot (version 1.29.1).

**Immunohistochemistry**

Immunohistochemistry was performed on 4 µm thick paraffin sections of human hippocampi using standard protocols (PMID 32196746). Antigen retrieval was performed with Tris-EDTA buffer (10 mM Tris, 1 mM EDTA, 0.05% Tween-20, pH 9) and citrate buffer (10 mM pH 6) for NFĸB and FoxO1 respectively. Primary antibodies were incubated in blocking solution over night at 4 °C (NFĸB, abcam, ab32360, 1:300; FoxO1, Cell Signaling, C29H4, 1:100: NeuN, Synaptic Systems, 266004, 1:200; GFAP, Sigma-Aldrich, G3893, 1:400; CD68, Abcam, ab9555, 1:100). After washing (PBS), respective secondary antibody (Alexa®Fluor A11011, A11001, A21450, 1:200) and DAPI (1:10.000) were incubated for 2 hours (h) at RT followed by washing and mounting (Mowiol).

**Multiplex Assay**

Multiplex Assay was performed on 4 µm thick paraffin sections of human hippocampi using the Akoya Fluorescent Multiplex kit (Akoya Biosciences) following the manufacturer’s protocol. Briefly, sections were deparaffinated followed by steaming antigen retrieval in retrieval buffer (pH 6) for 60 min using a household food steamer (Braun). Sections were blocked for 10 min with Opal Antibody Diluent/Block (Akoya Biosciences). Serially, primary antibodies against CD4 (Cell Signaling, 48274, 1:150), CD8 (Dako, M7103, 1:500), CD20 (ThermoScientific, MS-340, 1:2500), CD3 (Abcam, ab16667, 1:250), Iba1 (Wako, 019-19741, 1:15000), GFAP (Dako, Z0334, 1:15000), NeuN (Merck Millipore / Chemicon, MAB377, 1:2500), HuD (Proteintech, 14992-1-AP, 1:5000), CD103 (Abcam, ab129202, 1:5000) were incubated for 2 h at RT, followed by washing steps using Tris-buffered saline with Tween 20 (TBST). Opal polymer horseradish peroxidase (HRP) ms + Rb (PerkinElmer) was applied for 10 min at RT, followed by incubation with fluorophores (Opal 520, Opal 570, Opal 620, Opal 690, Opal 780). Sequential rounds involved fixation with 4% paraformaldehyde for 10 minutes at room temperature, followed by a repeated antigen retrieval step at pH 6.0 for 30 minutes before applying the next primary antibody. Finally, nuclei were stained with 4′,6-diamidino-2-phenylindole (DAPI).

**Reference list**

1. Zheng GX*, et al.* Massively parallel digital transcriptional profiling of single cells. *Nat Commun* **8**, 14049 (2017).

2. Hao Y*, et al.* Integrated analysis of multimodal single-cell data. *Cell* **184**, 3573-3587 e3529 (2021).

3. Alvarez M*, et al.* Enhancing droplet-based single-nucleus RNA-seq resolution using the semi-supervised machine learning classifier DIEM. *Sci Rep* **10**, 11019 (2020).

4. Tirosh I*, et al.* Dissecting the multicellular ecosystem of metastatic melanoma by single-cell RNA-seq. *Science* **352**, 189-196 (2016).

5. McGinnis CS, Murrow LM, Gartner ZJ. DoubletFinder: Doublet Detection in Single-Cell RNA Sequencing Data Using Artificial Nearest Neighbors. *Cell Syst* **8**, 329-337 e324 (2019).

6. Hafemeister C, Satija R. Normalization and variance stabilization of single-cell RNA-seq data using regularized negative binomial regression. *Genome Biol* **20**, 296 (2019).

7. Hulsen T. DeepVenn - a web application for the creation of area-proportional Venn diagrams using the deep learning framework Tensorflow.js. (2022).

8. Golomb SM*, et al.* Multi-modal Single-Cell Analysis Reveals Brain Immune Landscape Plasticity during Aging and Gut Microbiota Dysbiosis. *Cell Rep* **33**, 108438 (2020).

9. Cannon M*, et al.* DGIdb 5.0: rebuilding the drug-gene interaction database for precision medicine and drug discovery platforms. *Nucleic Acids Res* **52**, D1227-D1235 (2024).

10. Chen EY*, et al.* Enrichr: interactive and collaborative HTML5 gene list enrichment analysis tool. *BMC Bioinformatics* **14**, 128 (2013).

11. Duan Q*, et al.* L1000CDS(2): LINCS L1000 characteristic direction signatures search engine. *NPJ Syst Biol Appl* **2**, 16015- (2016).

12. Chen S, Zhou Y, Chen Y, Gu J. fastp: an ultra-fast all-in-one FASTQ preprocessor. *Bioinformatics* **34**, i884-i890 (2018).

13. Frankish A*, et al.* GENCODE reference annotation for the human and mouse genomes. *Nucleic Acids Res* **47**, D766-D773 (2019).

14. Patro R, Duggal G, Love MI, Irizarry RA, Kingsford C. Salmon provides fast and bias-aware quantification of transcript expression. *Nat Methods* **14**, 417-419 (2017).

15. Love MI*, et al.* Tximeta: Reference sequence checksums for provenance identification in RNA-seq. *PLoS Comput Biol* **16**, e1007664 (2020).

16. Leek JT, Johnson WE, Parker HS, Jaffe AE, Storey JD. The sva package for removing batch effects and other unwanted variation in high-throughput experiments. *Bioinformatics* **28**, 882-883 (2012).

17. Stephens M. False discovery rates: a new deal. *Biostatistics* **18**, 275-294 (2017).

18. Ritchie ME*, et al.* limma powers differential expression analyses for RNA-sequencing and microarray studies. *Nucleic Acids Res* **43**, e47 (2015).

19. Kanehisa M, Furumichi M, Sato Y, Ishiguro-Watanabe M, Tanabe M. KEGG: integrating viruses and cellular organisms. *Nucleic Acids Res* **49**, D545-D551 (2021).

20. Luo W, Brouwer C. Pathview: an R/Bioconductor package for pathway-based data integration and visualization. *Bioinformatics* **29**, 1830-1831 (2013).

21. Ge SX, Jung D, Yao R. ShinyGO: a graphical gene-set enrichment tool for animals and plants. *Bioinformatics* **36**, 2628-2629 (2020).

22. Eden E, Lipson D, Yogev S, Yakhini Z. Discovering motifs in ranked lists of DNA sequences. *PLoS Comput Biol* **3**, e39 (2007).

23. Eden E, Navon R, Steinfeld I, Lipson D, Yakhini Z. GOrilla: a tool for discovery and visualization of enriched GO terms in ranked gene lists. *BMC Bioinformatics* **10**, 48 (2009).

24. Ge SX, Son EW, Yao R. iDEP: an integrated web application for differential expression and pathway analysis of RNA-Seq data. *BMC Bioinformatics* **19**, 534 (2018).

25. Xie Z*, et al.* Gene Set Knowledge Discovery with Enrichr. *Curr Protoc* **1**, e90 (2021).

26. Neafsey PJ, Boxenbaum H, Ciraulo DA, Fournier DJ. A Gompertz age-specific mortality rate model of aging, hormesis, and toxicity: dose-response studies. *Drug Metab Rev* **20**, 111-150 (1989).

27. Keenan AB*, et al.* ChEA3: transcription factor enrichment analysis by orthogonal omics integration. *Nucleic Acids Res* **47**, W212-W224 (2019).
